# Hallmarks of Splicing Defects in Cancer: Clinical Applications in the Era of Personalized Medicine

**DOI:** 10.3390/cancers12061381

**Published:** 2020-05-28

**Authors:** Mohammad Alinoor Rahman, Farhana Nasrin, Sonali Bhattacharjee, Saikat Nandi

**Affiliations:** Cold Spring Harbor Laboratory, Cold Spring Harbor, NY 11724, USA; mrahman@cshl.edu (M.A.R.); nasrin@cshl.edu (F.N.)

**Keywords:** splicing, spliceosome, cancer therapies, oncogenesis, RNA processing

## Abstract

Alternative splicing promotes proteome diversity by using limited number of genes, a key control point of gene expression. Splicing is carried out by large macromolecular machineries, called spliceosome, composed of small RNAs and proteins. Alternative splicing is regulated by splicing regulatory *cis*-elements in RNA and *trans*-acting splicing factors that are often tightly regulated in a tissue-specific and developmental stage-specific manner. The biogenesis of ribonucleoprotein (RNP) complexes is strictly regulated to ensure that correct complements of RNA and proteins are coordinated in the right cell at the right time to support physiological functions. Any perturbations that impair formation of functional spliceosomes by disrupting the *cis*-elements, or by compromising RNA-binding or function of *trans*-factors can be deleterious to cells and result in pathological consequences. The recent discovery of oncogenic mutations in splicing factors, and growing evidence of the perturbed splicing in multiple types of cancer, underscores RNA processing defects as a critical driver of oncogenesis. These findings have resulted in a growing interest in targeting RNA splicing as a therapeutic approach for cancer treatment. This review summarizes our current understanding of splicing alterations in cancer, recent therapeutic efforts targeting splicing defects in cancer, and future potentials to develop novel cancer therapies.

## 1. Introduction

Humans and other higher metazoans have evolved by acquiring regulated diversity in their genes by inserting multiple noncoding introns into a coding region. Alternative splicing promotes proteome diversity without increasing the number of genes. With the advent of high-throughput sequencing, it is now evident that about 95% of human multi-exon genes undergo alternative splicing [1,2]. To support physiological and cellular functions, alternative splicing is controlled with high fidelity in tissue-specific, developmental stage-specific, and often gender-specific manners. It is further modulated in response to intracellular signals or external stimuli. Humans exploit the most complex alternative splicing regulation. The increasing complexity increases the possibility of splicing misregulation, which potentially affects physiological functions and gives rise to various human diseases, including cancers. The progress in high-throughput sequencing, and its application in sequencing human tumors versus matched normal tissues, have provided the opportunity to identify tumor-specific alternative splicing events. Splicing dysregulation can affect genes involved in virtually every step of the transformation process [3]. However, in the past several years, we have observed that many of these tumor-associated splicing changes arise due to alterations in particular components of the splicing machinery [4,5]. Therapeutic approaches to modulate splicing events in several genetic diseases are reaching the clinic. However, splicing modulation in cancer is still in progress. In this review, we discuss our current understanding of splicing alterations in cancer with mechanistic insights, highlight recent and ongoing strategies to target splicing defects in cancer, and examine future opportunities to develop novel cancer therapies.

## 2. Splicing Machinery, Splicing Code, and Splicing Catalysis

**Figure 1:**
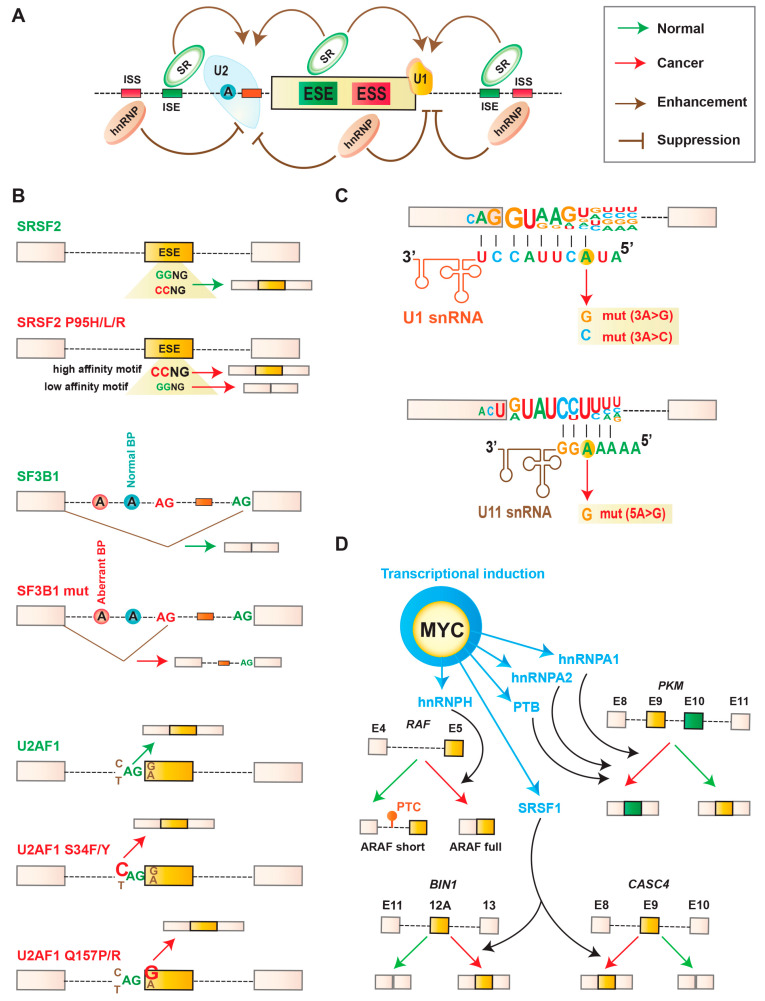
Splicing alterations in cancer. (**A**) Schematic of splicing regulatory *cis*-elements, which can influence alternative splicing regulation. Based on location and functional activity, these elements can be categorized into intronic/exonic splicing enhancers (ISEs/ESEs) and intronic/exonic splicing silencers (ISSs/ESSs). Exon inclusion or skipping is regulated by binding of cognate splicing *trans*-factors, such as serine/arginine (SR) proteins and heterogeneous nuclear ribonucleoproteins (hnRNPs). (**B**) Mechanistic consequences of cancer-associated recurrent mutations in spliceosomal genes *SRSF2*, *SF3B1*, and *U2AF1*. Mutations in Pro95 of SRSF2 changes its RNA-binding specificity from a G-rich motif (GGWG, W = A/U) to a C-rich motif ((C/G)CWG). Mutations in SF3B1 alter 3′ splice site by enhancing recognition of cryptic upstream 3′ splice sites. Mutations in U2AF1 alter 3′ splice site consensus sequences. Wild-type U2AF1 recognizes the consensus motif yAG|r at the intron–exon boundary (y = pyrimidine, r = purine, ‘|’ = intron–exon boundary). S34F or S34Y mutations promote recognition of cAG|r over tAG|r, whereas Q157P or Q157R mutations promote recognition of yAG|g over yAG|a. (**C**) Cancer-associated mutations in U1 small nuclear RNA (snRNA) and U11 snRNA. (**D**) Mechanistic consequences for abnormal expression of splicing-factor in cancer. MYC increases the expression of splicing factors SRSF1, hnRNPA1, hnRNPA2, PTB, and hnRNPH, which subsequently change the alternative splicing of downstream targets. SRSF1 promotes tumor specific splicing of *BIN1* and *CASC4.* HnRNPA1, A2, and PTB promote the expression of PKM2, a cancer-specific variant of PKM that induces aerobic glycolysis. HnRNPH promotes the expression of active oncogenic RAF (ARAF full) while repressing the short RAF isoform (ARAF short).

RNA splicing is a nuclear process catalyzed by large macromolecular machineries, known as the major (recognizes ~99.5% of introns) and minor spliceosome (recognizes ~0.5% of introns), composed of small RNAs and proteins [6]. Five small nuclear ribonucleoproteins (snRNPs) and multiple proteins (>100) cooperate to form the spliceosome. Each snRNP is composed of a single uridine-rich small nuclear RNA (snRNA) and multiple proteins. Splicing is accomplished in two steps: recognition of intron/exon boundary and catalysis of the transesterification reaction to excise out an intron followed by joining two exons. Recognition of intron/exon boundary is directed by essential splicing *cis*-elements present within the intron, termed as consensus splice site sequences. These include 5′ splice site, a branch point (BP), a polypyrimidine tract (PPT), and 3′ splice site. These splice sites are recognized by essential splicing factors. In humans and metazoans, these consensus splice site sequences are highly degenerative. Therefore, multiple auxiliary splicing factors cooperate to form functional spliceosome to promote splicing. The assembly of spliceosome is further coordinated by auxiliary splicing *cis*-elements: intronic/exonic splicing enhancers (ISEs/ESEs) and intronic/exonic splicing silencers (ISSs/ESSs) (Figure 1A). The majority of splicing *trans*-factors for ESEs are serine/arginine (SR)-rich proteins [7,8]. In contrast, the majority of splicing *trans*-factors for splicing silencer elements (ISSs/ESSs) are heterogeneous nuclear ribonucleoproteins (hnRNPs) [9]. SR proteins contain one or two RNA-recognition motif (RRM) at the N-terminal end and arginine and serine residues (arginine/serine-rich (RS) domains) at the C-terminal end. In contrast, hnRNPs usually contain an RRM-type and K homology (KH)-type RNA-binding domain along with other auxiliary domains.

Spliceosome assembly initiates with the recognition of the 5′ splice site by U1 snRNP, the BP by SF1, and the PPT as well as 3′ terminal AG by U2AF heterodimer (U2AF65 and U2AF35, respectively). This is an ATP-independent step, known as an E complex. An ATP-dependent spliceosome A complex is then formed, where SF1 is replaced by U2 snRNP at BP. Subsequent recruitment of U4/U6-U5 snRNPs leads to the formation of B complex. An active spliceosome complex called C complex is then formed by replacing U1 and U4 snRNPs, which subsequently catalyzes splicing. Generally, splicing activators or repressors modulate the early spliceosome assembly at the stage of E complex or A complex. Therefore, the ultimate splicing consequence is accomplished by complex *cis*-acting splicing code, their cognate RNA-binding *trans*-factors, and an enormous network of coordinated interactions.

## 3. Splicing Alterations in Cancer

Systematic and extensive sequencing of cancer versus normal tissues suggests that cancer cells display ‘noisier’ splicing compared to normal tissue [10]. It is now well established that changes in splicing affect every step of the oncogenic transformation. For example, tumor-specific increased expression of anti-apoptotic isoforms have been documented in *BCL2L1, CASP2,* and *FAS*; alternative splicing linked with the acquisition of invasive properties has been reported in *CD44, FGFR2, RAC1,* and *MST1R;* and angiogenesis-specific splice variants are reported in *VFGFA* [11]. Tumor-associated alterations in splicing occur either due to mutations in splicing regulatory *cis*-elements in RNA or changes in components of the splicing machinery. These alterations are further regulated in a tissue-specific or developmental stage-specific manner, and often also controlled by cellular signals or external stimuli.

### 3.1. Mutations in Splicing Regulatory Cis-Elements in RNA

Recent analyses of whole-exome, whole-genome, and RNA-sequencing of tumor and normal tissues from the same patients have provided important insights into the effects of somatic mutations in splicing alterations in cancer [12,13]. One critical event reported is intron retention, which happens more commonly than cassette exon splicing or alternative splice site selection and occurs in an allele-specific manner. Moreover, mutation-induced intron retention was exclusively enriched in tumor suppressor genes compared with oncogenes. Interestingly, most of the intron-retention events resulted in the generation of premature termination codons (PTCs) compared with cassette exon splicing events. The most commonly affected tumor suppressor genes included *TP53* (encodes p53), *ARID1A* (encodes a chromatin-remodeling factor), and *PTEN* (encodes a phosphatase regulating phosphatidylinositol 3-kinase (PI3K) signaling) [13]. This is also evident in several clinically important mutations that activate proto-oncogenes by altering critical splicing events. For instance, mutations in the proto-oncogene *MET* (encodes a receptor tyrosine kinase) promoted skipping of exon 14 in lung cancer [14,15,16]. In contrast, mutations in *NOTCH1* (which encodes a trans-membrane receptor) reported to activate a cryptic splice site and resulted in an aberrant isoform of NOTCH1 in chronic lymphocytic leukemia (CLL) [17]. In addition to intronic mutations affecting splice sites, a substantial proportion of somatic synonymous mutations have been reported within exons that affect ESE or ESS sequences [12]. Surprisingly, synonymous exonic mutations were enriched within oncogenes compared with tumor suppressor genes [12]. Furthermore, these synonymous mutations preferentially resulted in a gain of ESE motifs and a loss of ESS motifs, which is not usually observed for synonymous mutations in tumor suppressor genes [12].

### 3.2. Mutations in Components of the Splicing Machinery

In addition to mutations in splicing *cis*-elements as described above, numerous studies reported recurrent cancer-associated mutations in genes of splicing machinery components, consisting of splicing factors and small RNAs.

#### 3.2.1. Mutations in Splicing Factors

##### Change-of-Function Mutations

Genomic sequencing of the myeloid malignancies unexpectedly discovered recurrent heterozygous somatic mutations in several splicing factors [18]. Among these factors, SF3B1, SRSF2, and U2AF1, are most frequently mutated in patients with myelodysplastic syndrome (MDS), and also commonly occurred in clonal hematopoiesis, acute myeloid leukemia (AML), CLL, and a variety of solid tumors (Table 1) [18,19,20]. These mutations occur in highly restricted residues (hot spots) and are mutually exclusive (Table 1). Although initially it was predicted that these mutations might affect common downstream splicing targets, it is now evident that different mutations regulate hundreds of different splicing targets [4,5,21,22].

The splicing factor SRSF2 is a member of the SR-rich protein family. Cancer-associated recurrent mutations in SRSF2 predominantly occur at the Pro95 codon, which is in close proximity to the RRM domain [18]. These mutations in SRSF2 mutations show worse survival outcomes in MDS and an increased risk of transformation to AML [51]. Mutations in Pro95 of SRSF2 changes its RNA-binding specificity from a G-rich motif (GGWG, W = A/U) to a C-rich motif ((C/G)CWG) (Figure 1B) [52,53,54,55]. These gain-of-function mutations trigger genome-wide splicing alterations, affecting functions of many proteins, including important regulators associated with hematopoiesis. In addition to altered splicing, several mRNA isoforms promoted by mutant SRSF2 contain a PTC and are degraded by nonsense-mediated mRNA decay (NMD). Recently, it has been shown that mutations in SRSF2 also enhance its NMD-stimulating activity which is also promoted by sequence-specific RNA-binding and subsequent enhancement of exon junction complexes (EJCs) downstream from the PTC [55]. One notable example is *EZH2*, encoding enhancer of zeste homolog 2 protein, which catalyzes histone methylation and functions in chromatin remodeling. Pro95 mutation in SRSF2 promotes the inclusion of a poison exon in intron 8 of *EZH2*, resulting in a transcript with a PTC, which is degraded by NMD. Other important target genes include *INTS3* (a member of the integrator complex), *ARMC10* (tumor suppressing factor), *FYN* (the tyrosine kinase), *BCOR* (also recurrently mutated in AML and MDS), *IKAROS* (associated with the renewal of stem cell), and *CASP8* (a regulator of apoptosis) [52,53,54,55].

The splicing factor SF3B1 is a subunit of U2 snRNP, which promotes the stabilization of the U2 snRNP at the branch point during spliceosome assembly. Recurrent mutations in SF3B1 typically occur in the highly conserved C-terminal domain, between the fourth and eighth HEAT domain repeats [18,19]. About half of these missense mutations affect amino acid residue K700, whereas other mutations affect nearby hotspots (R625, H662, and K666) (Table 1). According to several studies, these mutations promote the activation of cryptic 3′ splice site (Figure 1B) [20,56,57,58,59,60,61,62]. These result in many mRNAs with a PTC, which are subsequently degraded by NMD. However, there is no evidence of a direct role of SF3B1 mutations in the NMD pathway to date. One notable altered spliced target in SF3B1 mutant cells is *ABCB7*, encoding the mitochondrial iron exporter protein. Aberrant usage of 3′ splice site in *ABCB7* causes retention of a 21-bp intronic segment, which generates a PTC and is subsequently degraded by NMD [58]. Some other dysregulated genes include *ALAS2*, *SLC25A37*, *ASXL1*, *CBL*, *CRNDE, TMEM14C, UQCC1*, etc. [20,56,57,58,59,60,61,62].

The splicing factor U2AF1 is a subunit of the U2 snRNP, which promotes recognition of the AG dinucleotide at the 3′ splice site (SS). Recurrent mutations in U2AF1 mostly affect residues at S34 or Q157, respectively, spanning two separate and conserved zinc finger domains (Table 1) [18,19]. U2AF1 mutations show worse survival and an increased risk of transformation to AML [51,63]. Mutations in S34 and Q157 differentially affect the recognition of 3′ splice site. S34 mutants promote recognition of 3′ SS bearing a C or A immediately preceding the AG (Figure 1B) [64,65,66,67,68]. In contrast, Q157 mutants promote recognition of 3′ SS harboring a G immediately downstream of AG (Figure 1B) [66]. Two notable targets in U2AF1 S34 mutant cells are H2AFY (encoding an H2A histone variant) and STRAP (encoding serine/threonine kinase receptor-associated protein) [68]. Some other reported target genes in U2AF1 mutant cells include *PICALM, MED24, GNAS, BCOR, KDM6A*, etc. [64,65,66,67,68,69,70,71,72].

As splicing factor mutations occur in a mutually exclusive manner, initially it was anticipated that these mutations might affect common downstream splicing targets. However, it is now established that each of the mutations affect different splicing targets. These disparate effects directed a search for convergent effects of these mutations in processes unrelated to splicing. To this end, it was revealed that mutations in U2AF1 [73] and SRSF2 [74] promote the formation of R-loops, which are defined as three-stranded structures composed of DNA–RNA hybrids. The increased R-loops in cells bearing mutations in SRSF2 or U2AF1 subsequently cause increased DNA damage and activation of the ATR (ataxia telangiectasia and Rad3-related protein) pathway. These data represent a novel effect of mutant U2AF1 and SRSF2 with important therapeutic implications. Going forward, it will be important to investigate whether mutant SF3B1 similarly impacts R-loop generation. Apart from R-loops, a recent report proposed that the U2AF1 S34F mutation affects interactions with the cleavage and polyadenylation (CP) machinery [75], which causes increased usage of a distal CP site and longer 3′ untranslated regions (UTRs). For example, altered CP of the mRNA encoding the autophagy ATG7 caused downregulation of ATG7, perturbed autophagy, and accumulation of secondary mutations. It will be important to determine whether other mutations in U2AF1 or SRSF2 or SF3B1also alter CP usage, 3′ UTR length, or autophagy.

In addition to the core spliceosome components described above, alterations in other RNA-binding proteins have also been reported in cancer, including hematological malignancies (such as MDS, de novo AML, or CLL). These mutations were reported in *PRPF8, SF3A1, LUCL7L2, SF1, U2AF2, HNRNPK, SRSF6, SRSF1, SRSF7, TRA2β, SRRM2, DDX1, DDX23, CELF4, HN*, *RBM10, SFPQ, PHF5A, HNRNPCL1, PCBP1, PCBP2, FUBP1, FUBP3, QKI,* etc. [76,77,78,79]. The existence of such mutations suggests that alterations in multiple steps of spliceosome assembly and splicing regulation can contribute to cancer development and pathogenesis.

##### Loss-of-Function Mutations

In contrast to recurrent change-of-function mutations in SRSF2, SF3B1, and U2AF1, in highly restricted residues or hot spots, ZRSR2 mutations in MDS are distributed throughout the gene [18]. These mutations span the X chromosome (Xp22.1) and frequently interrupt the coding sequence by directly or indirectly introducing in-frame stop codons. These mutations appear to be loss-of-function mutations, and mostly affect splicing of U12-type introns [80]. Examples of altered splicing events promoted by mutations in ZRSR2 include expression of MAPK (mitogen-activated protein kinases) pathway members and E2F transcription factors. Loss-of-function mutations have also been reported in the splicing factor RBM10. These mutations are associated with lung, bladder, and thyroid carcinomas [37,79,81]. RBM10 mutations identified in lung cancer cells disrupt splicing of NUMB, an inhibitor of NOTCH signaling, and promote cell growth [82]. Loss-of-function mutations reported in other splicing factors include *FUBP1, FUBP3, PCBP2, QKI,* etc. [79].

#### 3.2.2. Mutations in snRNA

Although recurrent somatic mutations in cancer are exclusively identified in protein-coding genes, very recently recurrent mutations in spliceosomal snRNAs have been reported in cancer patients, mostly in U1 snRNA, and a small percentage in U11 snRNA (Figure 1C) [83,84]. U1 snRNA functions in recognition of 5′ splice site. Two prominent mutations at base-position 3 in U1 snRNA were identified (3A > C and 3A > G), which span within the 5′ splice site binding region (Figure 1C) [83,84]. The 3A > C mutation was identified in medulloblastoma (MB), CLL, hepatocellular carcinoma (HCC), B-cell non-Hodgkin lymphoma (B-NHL), and pancreatic adenocarcinoma (PC). In contrast, the 3A > G mutation was reported exclusively to MB (most prominently in the Sonic hedgehog MB subtype). snRNA-mutant tumors display significant aberrant splicing, with an excess of cryptic 5′ splice site events [83,84]. Mutant U1-snRNA-promoted alternative splicing inactivates tumor suppressor genes (such as *PTCH1*) and activates oncogenes (such as *GLI2* and *CCND2*). Mutation in U11 snRNA was reported at base position 5 (5A > G), which also spans in the highly conserved region of 5′ splice site [83]. Mutations in snRNA exemplify a novel target for therapy and represent highly recurrent and tissue-specific mutations of non-protein coding genes in cancer.

#### 3.2.3. Abnormal Expression of Splicing Factors

Although recurrent mutations in splicing regulators have been frequently identified in hematologic malignancies, few such mutations have been detected in solid tumors [11]. This suggests a difference in splicing targets and/or splicing regulation in hematological malignancies compared to solid tumors. Interestingly, solid tumors display alterations in splicing factors, such as changes in levels, changes in gene copy number and/or changes in gene expression (Table 1). Some of these splicing factors exert oncogenic properties, whereas some show tumor suppressing activities. For example, SRSF1 [23,85], SRSF3 [25], SRSF6 [23,29], hnRNPA2/B1 [40], or hnRNPH [46], often display oncogenic properties. In contrast, RBM5, RBM6, RBM10 [82], or QKI [86], act as tumor suppressors. These RNA-binding proteins often regulate alternative splicing in a concentration-dependent manner. Therefore, relative changes in their expression level in the context of particular cancer types can alter global splicing regulation. These alternative splicing changes subsequently regulate many of the cellular processes known as “hallmarks” of cancer, such as cell proliferation, apoptosis, metabolism, invasion, and angiogenesis. However, the pathological consequences of these global splicing alterations are only beginning to be discovered. Here we will briefly discuss several examples.

The expression of splicing factors is often regulated by oncogenic signaling [11,85,87]. The transcription factor MYC, commonly amplified in cancers, induces the expression of several splicing factors, and subsequently contributes to altered splicing (Figure 1D). One of the earliest examples of the MYC-regulated splicing target was the glycolytic enzyme, pyruvate kinase (PKM). There are two mutually exclusive isoforms of PKM: PKM1 and PKM2. PKM1 is expressed in normal adult tissues and promotes oxidative phosphorylation. In contrast, PKM2, which is upregulated in many cancers, promotes aerobic glycolysis. MYC upregulates transcription of specific hnRNPs (hnRNPA1, hnRNPA2, and PTB) (Figure 1D). These alterations subsequently promote the expression of the PKM2 isoform and aerobic glycolysis in glioma (Figure 1D) [3,88].

Several SR proteins, such as SRSF1, SRSF3, and SRSF6, are also amplified in multiple cancer types (Table 1) [25,29,30]. SRSF1 is also reported as a direct transcriptional target of MYC [85,89]. It was shown that upregulated expression of SRSF1 promotes transformation of human and mouse mammary epithelial cells, which regulate splicing of hundreds of transcripts [23,85]. Some of the SRSF1-regulated splicing events include the *MST1R* (Ron) proto-oncogene and the kinases *MKNK2* and *S6K1*, which induce the expression of pro-oncogenic isoforms [23,85,90]. SRSF1 also regulates splicing of the apoptotic factor *BCL2L11* (*BIM*) generating the protein lacking pro-apoptotic activity [85]. In addition, SRSF1 regulates splicing of the tumor suppressor *BIN1* [85]. In breast cancer, SRSF1 promotes the inclusion of exon-9 of *CASC4* (Figure 1D). It has been reported that expression of the exon-9-included CASC4 isoform increases proliferation and decreases apoptosis, partially recapitulating SRSF1′s oncogenic effects [91]. In contrast to overexpression of SRSF1, downregulation of SRSF3 elicits p53-mediated cellular senescence, which is regulated via expression of p53β, an alternatively spliced isoform of p53 [92]. Another target of MYC is hnRNPH. Enhanced expression of hnRNPH regulates splicing of active oncogenic RAF (ARAF) kinase [93], increasing the expression of the long isoform (ARAF full) that promotes RAS-induced transformation (Figure 1D).

### 3.3. Post-Translational Modification of Splicing Factors

Post-translational modifications can alter the function of splicing factors or change the nuclear-cytoplasmic distribution. These modifications include phosphorylation, methylation, acetylation, sumoylation, etc. These modifications are regulated by signaling pathways. For example, the localization and functional activities of SR proteins are regulated by phosphorylation/dephosphorylation dynamics, mostly at serine residues within the RS domain. Phosphorylation of SRSF1 enhances protein–protein interactions with other splicing factors harboring the RS domain, such as U1–70K [94]. In contrast, dephosphorylation of SR or SR-related proteins promotes splicing catalysis [95,96]. The major regulators of SR protein phosphorylation include SR protein kinase family (SRPK), Clk/Sty kinase family and topoisomerase I [10,11,97]. Cancer-associated splicing events are often regulated via SR protein phosphorylation. For example, phosphorylation of SRSF1 and SRSF7 by AKT kinase promote growth-factor induced alternative splicing of the fibronectin EDA exon [98]. This effect was predicted to be an indirect regulation via SRPKs. Subsequently it was shown that SRPKs are indeed activated by AKT in response to EGF-signaling, which subsequently enhanced SRPK nuclear translocation and SR protein phosphorylation [99]. Several components of the AKT pathway have been reported to function as oncogenes or tumor suppressors. Overexpression of SRPK1 is also evident in multiple tumor types [100,101,102,103,104]. In addition to SR proteins, hnRNPs and other SR proteins are also regulated by post-translational modifications; for example, alternative splicing of a four-exon cassette in the CASPASE-9 gene [105,106]. The exon included isoform (CASPASE-9a) functions as a proapoptotic variant, whereas the exon excluded isoform (CASPASE-9b) acts as an antiapoptotic variant. Mechanistic investigation revealed that AKT-dependent phosphorylation of hnRNP L promotes exon skipping, and generates the antiapoptotic variant [105,106]. This subsequently promotes cell survival. In contrast, AKT-mediated phosphorylation of hnRNP A1 affects the translational activity, rather than splicing regulation. Phosphorylated hnRNP A1 is unable to induce IRES dependent translation of the CCND1 and c-MYC mRNAs [107].

## 4. Targeting Splicing Alterations for Cancer Therapies

As described above, it is now well established that cancer cells display widespread alterations in RNA splicing compared to normal cells. These findings suggest that modulating of RNA splicing factors by targeting specific transcripts, or even genome-wide, may have significant therapeutic potential. Here we discuss different therapeutic avenues of splicing modulation for cancer therapies, current progress, and future perspectives.

### 4.1. Targeting Core Spliceosome

**Figure 2:**
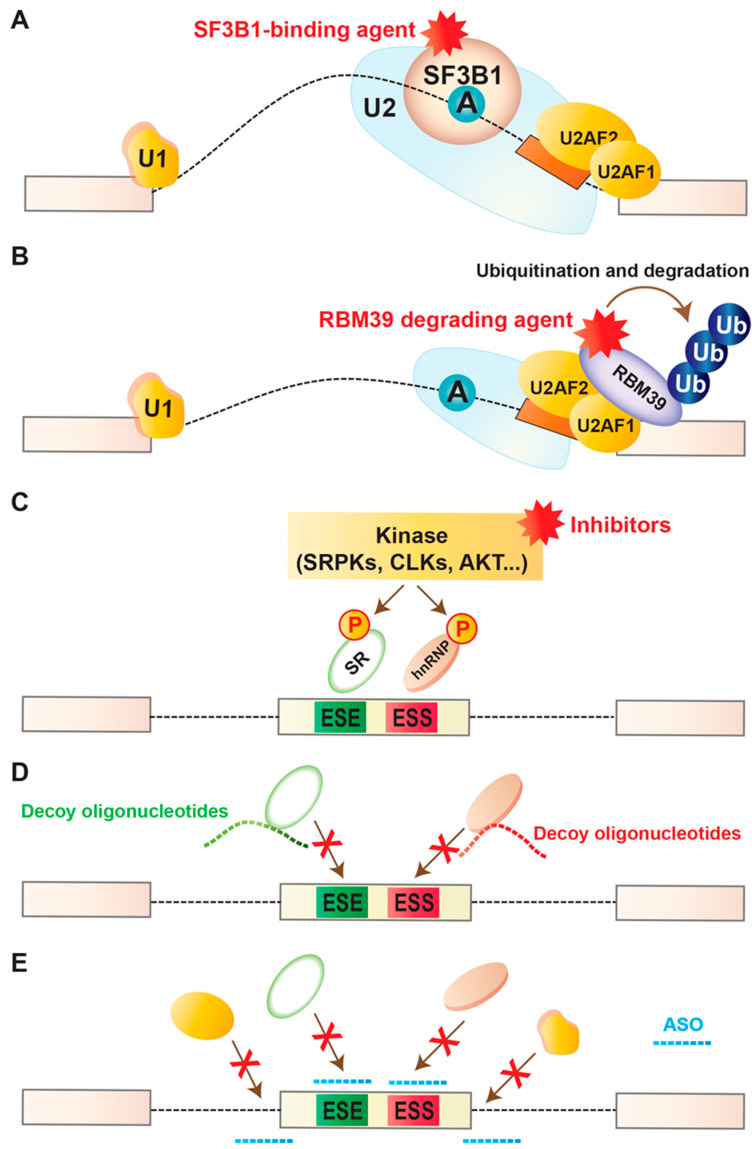
Therapeutic approaches of splicing modulation in cancer. (**A**) Pharmacological splicing inhibition using drugs that physically bind to the SF3B complex and disrupt its ability to recognize the branchpoint within the intron. See Table 2 for representative examples. (**B**) Anticancer sulfonamide compounds promote degradation of RBM39. These compounds physically connect RBM39 to the DCAF15-CUL4 ubiquitin ligase, resulting in ubiquitinylation of RBM39. This subsequently promotes proteasomal degradation of RBM39. See Table 2 for representative examples. (**C**) Pharmacological inhibition of post-translational modifications using drugs that interfere with the functions of several enzymes, including SR protein kinase family (SRPK), CDC2-like kinases (CLKs), and AKT kinases. See Table 2 for representative examples. (**D**) Decoy oligonucleotides containing short repeats of RNA-binding motifs can inhibit the function of specific splicing factor by sequestering them and prohibiting binding to the target transcript(s). One representative example was the transfection of specific decoy oligonucleotides into cancer cells targeting SRSF1-binding which resulted in changes in splicing of known targets of SRSF1, including *MKNK2* [134]. This subsequently activated the p38–MAPK stress pathway and inhibited the proliferation and survival of cancer cells [134]. (**E**) Antisense oligonucleotides (ASOs) can bind to splicing regulatory sequences or motifs in a targeted transcript, and switch splicing to a favorable isoform. One representative example was specific ASOs targeting a poison exon of BRD9 in SF3B1-mutant cells, which showed satisfactory results in splicing modulation and suppressing tumor growth [62].

In last two decades, multiple studies screened natural compounds for antitumor activity. Taken together, these led to the identification of several bacterially derived products. These can be categorized into three classes: pladienolides (such as pladienolides A–G); herboxidienes (such as GEX1A, 6-norherboxidiene), and spliceostatin (such as FR901463, FR901464, FR901465) (Table 2) [10,108,109]. Following the promising cytotoxic effects of these compounds in animal models, synthetic analogues were produced with improved chemical properties, including solubility, stability, potency etc. Among these analogues, noteworthy examples include E7107 (an analogue of pladienolide B), spliceostatin A (FR901464 derivative), meayamycin, sudemycin, etc. (Table 2). Although these compounds have different chemical structures, all of these drugs target the SF3B complex of the U2 snRNP (notably SF3B1 and PHF5A) and disrupt the early assembly of spliceosome (Figure 2A) [110,111,112,113]. This common effect drew promising attention for further investigation targeting the spliceosome for cancer therapy. Following the promising and selective antitumor activity of E7107 in human xenograft models, it was tested into two separate phase I clinical trials including 66 patients with locally advanced or metastatic solid tumors [114,115] (clinicaltrials.gov identifier NCT00499499). Although the tumors showed promising response in some patients, the visual side effects in a few patients precluded the clinical development of E7107 further. Whether the observed toxicity is a target-specific effect of SF3B1 inhibition, or an effect specifically associated with E7107, needs further elucidation. Further investigation is required to determine the safety margin and therapeutic efficacy of other structurally distinct forms of the pharmacologic compounds targeting the SF3B complex.

Note that SF3B complex is important for splicing, therefore general splicing inhibition has a high chance to be catastrophic for cells. Interestingly these drugs exert specific cytotoxicity to cancer cells [108]. The discovery of recurrent heterozygous mutations in components of spliceosome (such as SRSF2, SF3B1, and U2AF1) [18,135] emphasized the need to test these drugs for preferential sensitivity in spliceosomal-mutants. One notable fact is that cells with mutations in these genes retain expression of the wild-type allele and never become hemizygous. This suggests haplo essentiality of mutations in SRSF2, SF3B1, and U2AF1, and suggests that increased expression of the mutant allele is tightly regulated. E7107 treated isogenic murine myeloid leukemias showed preferential cell death of leukemia cells with mutated Srsf2 compared to wild-type Srsf2 [136]. A similar finding was observed for U2AF1 mutated cells treated with sudemycin [137]. However, there is no clinical evidence of the effects of SF3B inhibition on spliceosomal-mutant cancer. However, a phase I clinical trial of an orally administered SF3B inhibitior (H3B-8800) is currently ongoing in patients with AML, MDS, and chronic myelomonocytic leukemia (clinicaltrials.gov identifier NCT02841540). This trial will provide critical information about the safety margin of H3B-8800 in patients and efficiency of splicing modulation in patients. Further investigation in isogenic cancer cells with or without recurrent mutations will be necessary to understand the mechanistic insights of the pharmacological compounds whether they affect pre-mRNA *cis*-elements and/or aberrant protein(s) responsible for the preferential lethality in mutant cells.

### 4.2. Targeting Splicing Regulatory Proteins

#### 4.2.1. Sulfonamides

Several sulfonamide-containing compounds (such as indisulam, tasisulam, E7820, chloroquinoxaline) are known to show antitumor activity (Table 2). Several sulfonamides have already completed phase I and II clinical trials. For example, indisulam has been used in phase II clinical trials for patients with AML, melanoma, and non-small cell lung cancer with a satisfactory safety margin but limited efficacy [138,139,140]. The underlying mechanisms of how sulfonamide-containing compounds work were not known during the studies, which limited the opportunity to follow up the pharmacodynamic properties and mode of actions of these compounds. Two recent reports identified the U2AF-related splicing factor RBM39 as the target of several anticancer sulfonamide-containing compounds [123,124]. They showed that several sulfonamides promote ubiquitin-mediated degradation of RBM39 via CRL4 E3 ubiquitin ligase complex, known as DCAF15 (Figure 2B). It is important to note that RBM39 degradation was sensitive to a limited number of cancer cells [123,124]. RBM39 degradation interferes with splicing [141]. Interestingly, leukemia cells harboring splicing factor mutation showed similar sensitivity to RBM39 degradation similar to inhibition of SF3B complex [141], highlighting the promising therapeutic potentials of sulfonamides for spliceosomal-mutant cancer cells.

#### 4.2.2. Decoy Oligonucleotides

An attractive approach for targeting splicing regulatory proteins is via decoy oligonucleotides. These oligonucleotides are designed to contain short repeats of RNA-binding motifs targeting specific RNA-binding splicing factors in order to inhibit their function (Figure 2D) [134]. In a recent study, decoy oligonucleotides were developed targeting splicing factor SRSF1. As noted earlier, SRSF1 is frequently overexpressed in several different tumors, including lung, colon, breast, etc. [23,85,142]. SRSF1 overexpression affects global splicing regulation, promoting the expression of several pro-oncogenic isoforms, such as *MST1R* (Ron) proto-oncogene, and kinases like, *MKNK2* and *S6K1* [23,85,90]. Transfection of specific decoy oligonucleotides into cancer cells targeting SRSF1-binding resulted in changes in splicing of known targets of SRSF1, including *MKNK2* [134]. This subsequently activated the p38–MAPK stress pathway and inhibited the proliferation and survival of cancer cells [134]. This shows the promising potential of decoy oligonucleotide technology to treat cancers with upregulated or hyperactive splicing factors. However, one challenge behind this technology is several splicing factors with binding affinities for similar motifs might be sequestered; therefore, their downstream splicing targets could be aberrantly regulated and result in compromised biological functions. Careful considerations should be employed to test the feasibility and safety margin of this technology.

### 4.3. Targeting Post-Translational Modification

In addition to targeting spliceosome, efforts have been employed in developing drugs targeting post-translational modifications, an important regulation associated with spliceosome function. As noted earlier, post-translational modifications can alter the function of splicing factors or change the nuclear-cytoplasmic distribution. These modifications include phosphorylation, methylation, acetylation, sumoylation, etc., and are regulated by signaling pathways. Compounds with inhibiting properties of post-translational modifications have been reported to show promising anticancer effects (Figure 2C). One notable example is inhibition of phosphorylation of SR family proteins: SR proteins RRM at the N-terminal and an RS domain at the C-terminal [143,144]. The RS domains harbor multiple consecutive RS–SR dipeptide repeats, which are extensively phosphorylated by multiple kinases, including members of the SRPK family (SRPK1 and SRPK2), and the CDC2-like kinase (CLK) family (CLK1 to CLK4) [145,146]. Phosphorylation of SR proteins is often critical for the formation of spliceosome complex. In contrast, dephosphorylation promotes splicing catalysis to occur and promotes nuclear export of SR proteins. Screening of a series of chemical compounds or effects on in vitro phosphorylation of CLKs picked the benzothiazole compound TG-003 as a potential inhibitor of CLK1, CLK2, and CLK4 (Table 2) [129]. Although the global effects of TG-003 are still not well understood, the drug affects expression of the functional isoforms of CLK1 and SRSF2 [129]. Another oral treatment with the CLK inhibitor T-025 showed promising effects in cancer cells affecting alternative splicing, inducing cell death, and inhibiting cancer cell growth [130]. Screening for inhibitors of SRPK kinase activity identified Cpd-1, Cpd-2, and Cpd-3 [147]. These compounds inhibit SPRK1, SRPK2, and/or CLK1 and CLK2 [147]. Another example is SRPKIN-1, inhibitor of SRPK1 and SRPK2, which was reported to reduce SR protein phosphorylation, and subsequently promote the proangiogenic splicing isoform of VEGF (VEGF-A165a) to the anti-angiogenic VEGF- A165b isoform in a murine retinal model [126].

Another important example of post-translational modifications is arginine methylation. For example, the enzyme protein arginine *N*-methyltransferase 5 (PRMT5), which catalyzes symmetric dimethylation of arginine, is important for snRNP assembly and normal splicing [148,149]. Chemical inhibition of PRMT5 showed splicing inhibition and anticancer effects across a number of cancer types [150,151]. At least three PRMT5 inhibitors are now in phase I clinical trials for patients with relapsed/refractory solid tumors: GSK3326595 (clinicaltrials.gov NCT02783300); PF 06,939,999 (clinicaltrials.gov NCT03854227); JNJ-64619178 (clinicaltrials.gov NCT03573310). In contrast to PRMT5, PRMT1 catalyzes asymmetric dimethylation of arginine. PRMT1 is overexpressed in multiple cancers [152]. PRMT1 methylates splicing factor RBM15, triggering its ubiquitylation and subsequent degradation [153]. RBM15 is reported to bind to specific intronic sequences of genes encoding proteins with important roles in hematopoiesis [152]. Overexpression of PRMT1 reduces the expression of RBM15, and subsequently affects megakaryocyte terminal differentiation [152]. A phase I clinical trial with PRMT1 inhibitor is ongoing (GSK3368715) (clinicaltrials.gov NCT03666988). Combinatorial treatment with the PRMT1 and PRMT5 inhibitors MS023 and EPZ015666 was tested in a preclinical study, which showed efficient anticancer effects in small-cell lung cancer and pancreatic cancer cell lines [154].

### 4.4. Targeting MYC-Oncogene

Targeting MYC-onogene has significant therapeutic potentials for preferential splicing modulation in cancer. This is because MYC is the most frequently amplified oncogene in human cancers and plays a critical role in transformation. In addition, an important correlation between the MYC and the splicing machinery has been identified. Genes encoding several splicing regulatory proteins, such as *SRSF1*, *PTB*, *HNRNPA1*, and *HNRNPA2*, were reported to be direct transcriptional targets of MYC [3,85,89,91]. SRSF1 not only assists MYC’s oncogenic activity [89], but also contributes to its effects in transformation, promoting more aggressive forms of breast tumors by altering global splicing regulation [85,91]. On the other hand, hnRNPA1 and hnRNPA2 regulate alternative splicing of the cancer-associated muscle pyruvate kinase (PKM) isoform [3]. Recently it has been shown in B cell lymphomas that MYC directly upregulates several genes encoding core snRNP factors, and PRMT5 promotes the assembly of snRNP factors [155]. This study showed that downregulation of PRMT5 altered splicing, including exon skipping and intron retention, and inhibited lymphoma development in mice. This opens up a new avenue of therapeutic targeting by developing pharmacologic inhibitors of PRMT5, as described earlier. However, it is important to consider the fact that PRMT5 has diverse substrates apart from splicing factors. This fact is reflected in recent studies showing that cancer cells can be sensitized upon PRMT5 inhibition via other mechanisms, unrelated to RNA processing [156,157]. Further investigation is necessary to define the effects of pharmacologic inhibition of PRMT5 and its safety margin in MYC-dependent cancers. In an effort to seek the essential genes in MYC-overexpressing mammary epithelial cells, several splicing factors were identified, which are required to tolerate MYC hyperactivation. Among them, one important factor is BUD31, which is important for multiple subcomplexes of the spliceosome [150]. Downregulation of BUD31 resulted in global intron retention. Taken together these data suggest that the oncogenic activity of MYC is preferentially dependent on spliceosomal proteins, however, this is coordinated via differential mechanisms and is lineage specific. Therefore, the therapeutic efficacy of splicing modulation by targeting splicing factors and specificity for MYC-driven cancers should be further investigated.

### 4.5. Targeting mRNA Decay

Another approach is targeting other enzymatic steps in the mRNA decay pathway. One notable example is the N6-adenosine methyltransferase (METTL3) catalyzing N6-adenosine methylation of RNA, which is reported to be essential for the survival of certain cancer types, compared to healthy cells [125,158]. This observation has increased interests in developing chemical inhibitors of the METTL3 in cancer therapy [125,158]. Another potential target is the decapping enzyme scavenger DCPS. This enzyme catalyzes the final step of 3′ to 5′ mRNA decay. A recent study reported that DCPS is required for the survival of AML cells, although it is not essential for normal hematopoietic cells [159]. Several inhibitors of DCPS have been reported. One example is RG3039, which has completed a phase I clinical trial for spinal muscular atrophy (SMA) and appeared safe in patients [160]. This is therefore promising to assess the efficacy and safety margin of DCPS inhibitor in cancer therapy.

### 4.6. Targeted Splicing Modulation

An alternative and direct strategy is targeted modulation of a tumor-specific splicing event. One attractive approach to do this is by using antisense oligonucleotides (ASOs), which are short single-stranded nucleic acid molecules carrying different chemical modifications compared to RNA or DNA. ASOs can bind to splicing regulatory sequences or motifs in a targeted transcript, and switch splicing to a favorable isoform (Figure 2E) [161,162]. The target sequences can be splice sites (5′ SS or 3′ SS), silencers (ISS or ESS), or enhancers (ISE or ESE), which are normally recognized by the core splicing machinery or by RNA-binding splicing regulatory protein(s) (activator or suppressor). Therefore, antisense compounds can be designed to activate or inhibit a splicing event in a transcript-specific manner. Due to the design against unique complementary sequences in the transcriptome, this approach is efficient and expected to raise minimal off-target effects. The biggest challenge is targeting key splicing events, which are essential for oncogenic transformation or maintenance of tumors, which requires extensive studies and careful validations. Despite these challenges, ASO therapies are already in the clinic, and many therapeutic ASOs against several diseases, including cancer, are now in clinical trials [162,163,164]. For example, the ASO drug Nusinersen (Spinraza) corrects aberrant splicing of *SMN2* and is an effective approved treatment for spinal muscular atrophy [162,165]. Nusinersen binds to an intronic region flanking exon 7 in *SMN2* pre-mRNA, encoding survival motor neuron protein, and represses the recognition of exon 7 by the spliceosome. This subsequently enhances the inclusion of exon 7 in the mRNA, and encodes a functional full-length protein, which is normally lacking in spinal muscular atrophy due to homozygous mutations or deletions of the *SMN1* gene [162,165,166]. Another example is the ASO drug Eteplirsen for the treatment of Duchenne muscular dystrophy [164]. Eteplirsen binds to a site in exon 51 of the *DMD* pre-mRNA encoding dystrophin, and sterically blocks the recognition of exon 51 by the spliceosome. This subsequently promotes skipping of exon 51 and rectifies the disease-causing frameshift mutation [164]. The resulting mRNA generates a short but functional protein.

ASO drugs in cancer therapy are still in developmental stages, with promising results in several preclinical studies. One noteworthy example is the PKM gene which involves a choice between mutually exclusive exons 9 and 10. PKM1 isoform includes exon 9 and promotes oxidative phosphorylation. In contrast, the PKM2 isoform includes exon 10 and promotes aerobic glycolysis (the Warburg effect) and is crucial for tumor growth. The PKM2 isoform is frequently expressed in cancers. ASOs that impaire the expression of PKM2 elicit apoptosis in glioblastoma cell lines [167]. Recently, the antisense approach has been tested in the context of spliceosomal-mutant cancer. As noted earlier, SF3B1 is the most commonly mutated RNA splicing factor in cancer. Diverse SF3B1 mutations converge on repression of a critical target BRD9 [62], which is a core component of the non-canonical BAF chromatin-remodeling complex. Mutant SF3B1 recognizes an aberrant, deep intronic branchpoint within BRD9, and thereby promotes the inclusion of a poison exon [62]. This subsequently elicits degradation of BRD9 mRNA via NMD [62]. Depletion of BRD9 causes the loss of non-canonical BAF complex and promotes melanomagenesis. BRD9 is a potent tumor suppressor in uveal melanoma. In an attempt to correct mis-splicing of BRD9, specific ASOs were employed both in vitro and in vivo, which showed satisfactory results in splicing modulation and suppressing tumor growth [62].

In addition to ASOs, targeted modulation of a tumor-specific splicing event can also be achieved using small molecules. For example, several studies identified compounds that enhance *SMN2* exon 7 splicing to promote the production of a full-length SMN protein [102,168,169], which is promising for therapeutic utility for spinal muscular atrophy. Similarly, another study identified small molecules that promote the inclusion of exon 20 in *IKBKAP* pre-mRNA, which has shown promising results in familial dysautonomia [170]. These molecules were initially screened using reporters in a model cell. Although potential mechanisms have been proposed, further work is required to evaluate target specificity and safety margin, and generalized application to other splicing targets.

## 5. Perspective

Splicing alterations in human tumors are a growing area of interest in cancer research and are prospective targets for personalized cancer therapies. The advent of RNA-seq in recent years has provided extensive information to identify altered splicing targets in cancer. Analysis of aberrant splicing has not only uncovered the underlying maladies in cancer, but also allowed us to gain insight on the physiological regulation of splicing. Although these studies have provided important primary insights, we lack adequate information necessary to develop molecular therapies. One important impediment is to screen out critical “driver splicing targets” among hundreds of splicing changes, which are just the outcome of mutations or abnormal expression of splicing factors and not directly associated with the disease, also referred as “passenger splicing events”. In addition, it is difficult to ascertain the contribution of individual splicing events in the context of cancer. Moreover, the extent of splicing changes are often variable among clinical patients and model cell lines, making it difficult to target a promising splicing event for therapeutic correction. Systematic evaluation of the functional roles of tumor-specific RNA isoforms will be greatly instructive in developing targeted therapies. As we start understanding the biological consequences of splicing factor alterations in human tumors, we realize that many of these splicing changes are cell-type-specific. Further efforts are therefore necessary to dissect the precise roles of splicing factors in normal tissue and the consequences of their dysregulation in the context of cancer with adequate resolution at the proteome level. A better understanding of the cell-type specificity and functions of cancer-associated splicing factors will be critical to identify novel biomarkers and develop new therapeutic strategies.

A number of chemical compounds have been reported that modulate splicing; however, most of these drugs interfere with early spliceosome assembly (such as compounds targeting SF3B1) or phosphorylation of splicing factors (such as SR proteins). Further efforts are necessary to screen compounds targeting later stages of spliceosome assembly, which may be crucial in developing effective splicing modulating drugs. Recent advances on the structural understanding of the spliceosome will strongly help elucidate the mechanistic effects of these compounds and may help discover novel means of splicing modulation. While identifying new therapeutic modalities targeting splicing, it is important to consider the therapeutic index, safety margin, and toxicity effects of splicing modulation in patients. Systematic studies should carefully evaluate the feasibility of globally modifying RNA splicing in patients. It is plausible that the widespread alterations in splicing that result from targeting spliceosome assembly using chemical compounds may generate novel protein isoform(s), which could be exploited in immunologically targeting cancer cells. Therefore, systematic evaluation of the effects of these compounds at the proteome level is critical to expand our understanding of tumor pathogenesis and to identify more effective drug candidates. Cutting-edge technologies, such as splicing and proteomic profiling within single cells and single-molecule RNA-seq could be applied to gain novel insights into splicing dysregulation in cancer.

The identification of functionally important pathologic RNA isoforms opens up the possibility of antisense therapies for splicing alterations in cancer. Although antisense approaches to modulate splicing in several human genetic diseases are reaching the clinic, they are still in developmental stages in cancer therapies. More studies need to scrutinize antisense approaches for targeted and personalized cancer therapies. Another recently emerging area in cancer therapy is targeting neoepitopes generated by tumor-specific alternative splicing, which can be exploited to induce T cell responses in cancer patients. Systematic detection of immunogenic neoantigens is required for the successful development of potent vaccines. Recent studies show promising results in melanoma by expanding neoantigen-specific T cell populations [171,172]. Despite the major advances toward targeted cancer therapies, one critical challenge is the development of resistance to such treatments. To address this challenge, studies are required to define the underlying molecular mechanisms that drive tumorigenesis and links to distinct cellular and extracellular pathways that contribute to cancer phenotypes, which will be crucial for the development of more effective drugs [32,173,174,175,176,177,178,179,180].

## Figures and Tables

**Table 1:** Alterations in splicing factor in cancer.

Gene	Alteration	Tumor Type	Reference
*SRSF2*	Mutation	MDS, AML, CMML, RARS, RCMD-RS, MPN, UVM	[4,11,18,19]
	Hot spot: P95		
	Overexpression	Ovary	
*SF3B1*	Mutation	MDS, AML, CMML, CLL, RARS, RCMD-RS, MPN, UVM, MM, Breast, PDAC	[4,11,18,19]
	Hotspot: K700, E622, R625, H662, K666		
*U2AF1*	Mutation	MDS, AML, CMML, RARS, RCMD-RS, MPN, Lung	[4,11,18,19]
	Hot spot: S34, Q157		
*ZRSR2*	Mutation	MDS, AML, CMML, RARS, RCMD-RS, MPN	[4,11,18,19]
	No hot spot		
*SRSF1*	Amplification, overexpression	Breast, lung, colon, ovary, thyroid kidney, small intestine	[11,23,24]
*SRSF3*	Downregulation	Liver	[11,25,26,27]
	Amplification, overexpression	Ovary, cervix, breast, skin, stomach, bladder, thyroid, kidney, colon, liver	
*SRSF5*	Overexpression	Breast	[28]
*SRSF6*	Amplification, overexpression	Breast, Lung, colon, skin	[23,29,30]
*SRSF10*	Overexpression	Colorectal	[31]
*TRA2B*	Overexpression	Breast, colon, glioblastoma, ovary	[11,24,32,33]
*RBM5*	Downregulation	Lung, prostate	[34,35,36]
	Overexpression	Breast	
*RBM10*	Mutation	Lung	[34,37]
	Overexpression	Breast	
*ESRP1, ESRP2*	Overexpression/downregulation	Breast, oral	[38,39]
*HNRNPA1*	Overexpression	Glioblastoma, breast, colon, lung	[23,40,41,42]
*HNRNPA2/B1*	Amplification, overexpression	Glioblastoma	[40]
*HNRNPM*	Overexpression	Breast	[43]
*HNRNPK*	Downregulation	AML	[44,45]
	Overexpression	Breast, pancreatic, skin, esophageal, colorectal, oral	
*HNRNPH*	Overexpression	Glioblastoma	[46]
*PTB*	Overexpression	Breast, ovary, oral	[27,47,48]
*QKI*	Downregulation	Lung adenocarcinoma, prostate, oral	[49,50]

Note: MDS, myelodysplastic syndrome; AML, acute myeloid leukemia; CMML, chronic myelomonocytic leukemia; RARS, refractory anemia with ringed sideroblasts; RCMD-RS, refractory cytopenia with multilineage dysplasia with ringed sideroblasts; MPN, myeloproliferative neoplasms; UVM, uveal melanoma; CLL, chronic lymphocytic leukemia; MM, mucosal melanoma; PDAC, pancreatic ductal adenocarcinoma.

**Table 2:** Small molecule splicing modulators in cancer.

Compound	Target of Inhibition	Mechanism(s) of Action	Reference	
Pladienolide:	SF3B complex	Interact with SF3B1 and promote conformational alteration	[10,116,117,118]
Pladienolides A–G		Antitumor activities reported in human BSY-1, PC-3, OVCAR-3, DU-145, WiDr, HCT-116 cells, primary human colon cancer cells, xenograft mouse model, etc.	
Pladienolide analog:	SF3B complex	Interacts with SF3B1 and promotes splicing alteration	[10,118,119,120]
E7107		Inhibition of tumor growth in human breast, ovary, non-small cell lung cancer, xenograft mouse model, etc.	
Spliceostatin:	SF3B complex	Interface interaction of U2 snRNP with pre-	[10,118,121,122]
FR901463			
FR901464		mRNA	
FR901465			
Meayamycin B		Antitumor activities reported in murine solid tumors (colon 38 and Meth A), human lung adenocarcinoma solid tumor (A549), human colorectal carcinoma (HCT-116), human prostate tumor (PC-3), human rhabdomyosarcoma (rh18), xenograft mouse model, etc.	
Spliceostatin A			
Sudemycins			
Sufonamides:	RBM39	Bind a receptor of the CRL4 E3 ubiquitin ligase complex (known as DCAF15), and direct ubiquitin-mediated degradation of RBM39	[123,124,125]
Indisulam			
Tasisulam			
E7820			
SRPK inhibitor:	SRPK1 and SRPK2	Competitively inhibit SRPK1 and SRPK2, subsequently affect SR protein phosphorylation	[126,127,128]
SRPIN340			
SRPKIN-1		SRPIN340 is reported to block angiogenesis and related tumor growth in nude mice	
		SRPKIN-1 is reported to convert the proangiogenic VEGF isoform to the angiogenic isoform	
CLK inhibitor:	CLK1, CLK2, CLK4	Competitively inhibit CLK1, CLK2, and CLK4, and subsequently affect SR protein phosphorylation	[10,129,130]
T-025			
TG003		T-025 is reported to affect global alternative splicing, inducing cancer cell death, and inhibiting cancer cell growth	
SRPK and CLK	SRPK1, SRPK2, CLK1, CLK2	Inhibit SRPK1, SRPK2, CLK1, and CLK2, and subsequently affect SR protein phosphorylation	[10,118,131]
inhibitor:			
Cpd-1			
Cpd-2			
Cpd-3			
Topoisomerase I inhibitor:	Topoisomerase I	Inhibitory effect prior to step one of splicing	[132]
NB-506		Inhibits SRSF1 phosphorylation and splicing of Bcl-X, CD44, SRSF2, and Sty in P388 cultured cells	
Amiloride	Unknown	Inhibits SRSF1 phosphorylation and splicing of Bcl-X, HIPK3, and RON/MISTR1 in Huh-7 cultured cells	[133]

## Abbreviations

AML	Acute myeloid leukemia
ASO	Antisense oligonucleotides
ATR	Ataxia telangiectasia and Rad3-related protein
B-NHL	B-cell non-Hodgkin lymphoma
BP	Branch point
CLK	CDC2-like kinase
CLL	Chronic lymphocytic leukemia
CP	Cleavage and polyadenylation
EJCs	Exon junction complexes
ESEs	Exonic splicing enhancers
ESSs	Exonic splicing silencers
HCC	Hepatocellular carcinoma
hnRNPs	Heterogeneous nuclear ribonucleoproteins
ISEs	Intronic splicing enhancers
ISSs	Intronic splicing silencers
MB	Medulloblastoma
MDS	Myelodysplastic syndrome
METTL3	N6-adenosine methyltransferase
NMD	Nonsense-mediated mRNA decay
PC	Pancreatic adenocarcinoma
PPT	Polypyrimidine tract
PTCs	Premature termination codons
PRMT5	Protein arginine *N*-methyltransferase 5
PKM	Pyruvate kinase
RNPs	Ribonucleoproteins
RRM	RNA-recognition motif
SRPK	SR protein kinase family
snRNPs	Small nuclear ribonucleoproteins
SS	Splice site

## References

[1] Pan Q., Shai O., Lee L.J., Frey B.J., Blencowe B.J. (2008). Deep surveying of alternative splicing complexity in the human transcriptome by high-throughput sequencing. Nat. Genet..

[2] Wang E.T., Sandberg R., Luo S., Khrebtukova I., Zhang L., Mayr C., Kingsmore S.F., Burge C.B. (2008). Alternative isoform regulation in human tissue transcriptomes. Nature.

[3] David C.J., Chen M., Assanah M., Canoll P., Manley J.L. (2010). HnRNP proteins controlled by c-Myc deregulate pyruvate kinase mRNA splicing in cancer. Nature.

[4] Dvinge H., Kim E., Abdel-Wahab O., Bradley R.K. (2016). RNA splicing factors as oncoproteins and tumour suppressors. Nat. Rev. Cancer.

[5] Rahman M.A., Krainer A.R., Abdel-Wahab O. (2020). SnapShot: Splicing alterations in cancer. Cell.

[6] Wahl M.C., Will C.L., Lührmann R. (2009). The spliceosome: Design principles of a dynamic RNP machine. Cell.

[7] Graveley B.R. (2000). Sorting out the complexity of SR protein functions. RNA.

[8] Lin S., Fu X.D. (2007). SR proteins and related factors in alternative splicing. Adv. Exp. Med. Biol..

[9] Martinez-Contreras R., Cloutier P., Shkreta L., Fisette J.F., Revil T., Chabot B. (2007). hnRNP proteins and splicing control. Adv. Exp. Med. Biol..

[10] Lee S.C., Abdel-Wahab O. (2016). Therapeutic targeting of splicing in cancer. Nat. Med..

[11] Anczuków O., Krainer A.R. (2016). Splicing-factor alterations in cancers. RNA.

[12] Supek F., Miñana B., Valcárcel J., Gabaldón T., Lehner B. (2014). Synonymous mutations frequently act as driver mutations in human cancers. Cell.

[13] Jung H., Lee D., Lee J., Park D., Kim Y.J., Park W.Y., Hong D., Park P.J., Lee E. (2015). Intron retention is a widespread mechanism of tumor-suppressor inactivation. Nat. Genet..

[14] Ma P.C., Kijima T., Maulik G., Fox E.A., Sattler M., Griffin J.D., Johnson B.E., Salgia R. (2003). c-MET mutational analysis in small-cell lung cancer: Novel juxtamembrane domain mutations regulating cytoskeletal functions. Cancer Res..

[15] Cancer Genome Atlas Research Network (2012). Comprehensive genomic characterization of squamous cell lung cancers. Nature.

[16] Cancer Genome Atlas Research Network (2014). Comprehensive molecular profiling of lung adenocarcinoma. Nature.

[17] Puente X.S., Beà S., Valdés-Mas R., Villamor N., Gutiérrez-Abril J., Martín-Subero J.I., Munar M., Rubio-Pérez C., Jares P., Aymerich M. (2015). Noncoding recurrent mutations in chronic lymphocytic leukemia. Nature.

[18] Yoshida K., Sanada M., Shiraishi Y., Nowak D., Nagata Y., Yamamoto R., Sato Y., Sato-Otsubo A., Kon A., Nagasaki M. (2011). Frequent pathway mutations of splicing machinery in myelodysplasia. Nature.

[19] Papaemmanuil E., Gerstung M., Malcovati L., Tauro S., Gundem G., Van Loo P., Yoon C.J., Ellis P., Wedge D.C., Pellagatti A. (2013). Clinical and biological implications of driver mutations in myelodysplastic syndromes. Blood.

[20] Darman R.B., Seiler M., Agrawal A.A., Lim K.H., Peng S., Aird D., Bailey S.L., Bhavsar E.B., Chan B., Colla S. (2015). Cancer-Associated SF3B1 Hotspot Mutations Induce Cryptic 3’ Splice Site Selection through Use of a Different Branch Point. Cell Rep..

[21] Rahman M.A., Nasrin F. (2016). Human Disease-causing mutations affecting RNA splicing and NMD. J. Investig. Genomics.

[22] Rahman M.A. (2018). Oncogenic Landscapes of Splicing-Factor Mutant MDS. Genom. Gene. Ther. Int. J..

[23] Karni R., de Stanchina E., Lowe S.W., Sinha R., Mu D., Krainer A.R. (2007). The gene encoding the splicing factor SF2/ASF is a proto-oncogene. Nat. Struct. Mol. Biol..

[24] Watermann D.O., Tang Y., Zur Hausen A., Jäger M., Stamm S., Stickeler E. (2006). Splicing factor Tra2-β1 is specifically induced in breast cancer and regulates alternative splicing of the *CD44* gene. Cancer Res..

[25] Jia R., Li C., McCoy J.P., Deng C.X., Zheng Z.M. (2010). SRp20 is a proto-oncogene critical for cell proliferation and tumor induction and maintenance. Int. J. Biol. Sci..

[26] Sen S., Langiewicz M., Jumaa H., Webster N.J. (2015). Deletion of serine/arginine-rich splicing factor 3 in hepatocytes predisposes to hepatocellular carcinoma in mice. Hepatology.

[27] He X., Ee P.L., Coon J.S., Beck W.T. (2004). Alternative splicing of the multidrug resistance protein 1/ATP binding cassette transporter subfamily gene in ovarian cancer creates functional splice variants and is associated with increased expression of the splicing factors PTB and SRp20. Clin. Cancer Res..

[28] Huang C.S., Shen C.Y., Wang H.W., Wu P.E., Cheng C.W. (2007). Increased expression of SRp40 affecting CD44 splicing is associated with the clinical outcome of lymph node metastasis in human breast cancer. Clin. Chim. Acta.

[29] Cohen-Eliav M., Golan-Gerstl R., Siegfried Z., Andersen C.L., Thorsen K., Orntoft T.F., Mu D., Karni R. (2013). The splicing factor SRSF6 is amplified and is an oncoprotein in lung and colon cancers. J. Pathol..

[30] Jensen M.A., Wilkinson J.E., Krainer A.R. (2014). Splicing factor SRSF6 promotes hyperplasia of sensitized skin. Nat. Struct. Mol. Biol..

[31] Zhou X., Li X., Cheng Y., Wu W., Xie Z., Xi Q., Han J., Wu G., Fang J., Feng Y. (2014). BCLAF1 and its splicing regulator SRSF10 regulate the tumorigenic potential of colon cancer cells. Nat. Commun..

[32] Fischer D.C., Noack K., Runnebaum I.B., Watermann D.O., Kieback D.G., Stamm S., Stickeler E. (2004). Expression of splicing factors in human ovarian cancer. Oncol. Rep..

[33] Iborra S., Hirschfeld M., Jaeger M., Zur Hausen A., Braicu I., Sehouli J., Gitsch G., Stickeler E. (2013). Alterations in expression pattern of splicing factors in epithelial ovarian cancer and its clinical impact. Int. J. Gynecol. Cancer.

[34] Rintala-Maki N.D., Goard C.A., Langdon C.E., Wall V.E., Traulsen K.E., Morin C.D., Bonin M., Sutherland L.C. (2007). Expression of RBM5-related factors in primary breast tissue. J. Cell Biochem..

[35] Oh J.J., West A.R., Fishbein M.C., Slamon D.J. (2002). A candidate tumor suppressor gene, H37, from the human lung cancer tumor suppressor locus 3p21.3. Cancer Res..

[36] Zhao L., Li R., Shao C., Li P., Liu J., Wang K. (2012). 3p21.3 tumor suppressor gene RBM5 inhibits growth of human prostate cancer PC-3 cells through apoptosis. World J. Surg. Oncol..

[37] Imielinski M., Berger A.H., Hammerman P.S., Hernandez B., Pugh T.J., Hodis E., Cho J., Suh J., Capelletti M., Sivachenko A. (2012). Mapping the hallmarks of lung adenocarcinoma with massively parallel sequencing. Cell.

[38] Yae T., Tsuchihashi K., Ishimoto T., Motohara T., Yoshikawa M., Yoshida G.J., Wada T., Masuko T., Mogushi K., Tanaka H. (2012). Alternative splicing of CD44 mRNA by ESRP1 enhances lung colonization of metastatic cancer cell. Nat. Commun..

[39] Ishii H., Saitoh M., Sakamoto K., Kondo T., Katoh R., Tanaka S., Motizuki M., Masuyama K., Miyazawa K. (2014). Epithelial splicing regulatory proteins 1 (ESRP1) and 2 (ESRP2) suppress cancer cell motility via different mechanisms. J. Biol. Chem..

[40] Golan-Gerstl R., Cohen M., Shilo A., Suh S.S., Bakacs A., Coppola L., Karni R. (2011). Splicing factor hnRNP A2/B1 regulates tumor suppressor gene splicing and is an oncogenic driver in glioblastoma. Cancer Res..

[41] Zhou J., Allred D.C., Avis I., Martinez A., Vos M.D., Smith L., Treston A.M., Mulshine J.L. (2001). Differential expression of the early lung cancer detection marker, heterogeneous nuclear ribonucleoprotein-A2/B1 (hnRNP-A2/B1) in normal breast and neoplastic breast cancer. Breast Cancer Res. Treat..

[42] Fielding P., Turnbull L., Prime W., Walshaw M., Field J.K. (1999). Heterogeneous nuclear ribonucleoprotein A2/B1 up-regulation in bronchial lavage specimens: A clinical marker of early lung cancer detection. Clin. Cancer Res..

[43] Xu Y., Gao X.D., Lee J.H., Huang H., Tan H., Ahn J., Reinke L.M., Peter M.E., Feng Y., Gius D. (2014). Cell type-restricted activity of hnRNPM promotes breast cancer metastasis via regulating alternative splicing. Genes Dev..

[44] Carpenter B., McKay M., Dundas S.R., Lawrie L.C., Telfer C., Murray G.I. (2006). Heterogeneous nuclear ribonucleoprotein K is over expressed, aberrantly localised and is associated with poor prognosis in colorectal cancer. Br. J. Cancer.

[45] Zhou R., Shanas R., Nelson M.A., Bhattacharyya A., Shi J. (2010). Increased expression of the heterogeneous nuclear ribonucleoprotein K in pancreatic cancer and its association with the mutant p53. Int. J. Cancer.

[46] Lefave C.V., Squatrito M., Vorlova S., Rocco G.L., Brennan C.W., Holland E.C., Pan Y.X., Cartegni L. (2011). Splicing factor hnRNPH drives an oncogenic splicing switch in gliomas. EMBO J..

[47] Jin W., McCutcheon I.E., Fuller G.N., Huang E.S., Cote G.J. (2000). Fibroblast growth factor receptor-1 α-exon exclusion and polypyrimidine tract-binding protein in glioblastoma multiforme tumors. Cancer Res..

[48] Takahashi H., Nishimura J., Kagawa Y., Kano Y., Takahashi Y., Wu X., Hiraki M., Hamabe A., Konno M., Haraguchi N. (2015). Significance of polypyrimidine tract-binding protein 1 expression in colorectal cancer. Mol. Cancer Ther..

[49] Lu W., Feng F., Xu J., Lu X., Wang S., Wang L., Lu H., Wei M., Yang G., Wang L. (2014). QKI impairs self-renewal and tumorigenicity of oral cancer cells via repression of SOX2. Cancer Biol. Ther..

[50] Zhao Y., Zhang G., Wei M., Lu X., Fu H., Feng F., Wang S., Lu W., Wu N., Lu Z. (2014). The tumor suppressing effects of QKI-5 in prostate cancer: A novel diagnostic and prognostic protein. Cancer Biol. Ther..

[51] Thol F., Kade S., Schlarmann C., Löffeld P., Morgan M., Krauter J., Wlodarski M.W., Kölking B., Wichmann M., Görlich K. (2012). Frequency and prognostic impact of mutations in SRSF2, U2AF1, and ZRSR2 in patients with myelodysplastic syndromes. Blood.

[52] Kim E., Ilagan J.O., Liang Y., Daubner G.M., Lee S.C.W., Ramakrishnan A., Li Y., Chung Y.R., Micol J.B., Murphy M.E. (2015). SRSF2 Mutations Contribute to Myelodysplasia by Mutant-Specific Effects on Exon Recognition. Cancer Cell.

[53] Zhang J., Lieu Y.K., Ali A.M., Penson A., Reggio K.S., Rabadan R., Raza A., Mukherjee S., Manley J.L. (2015). Disease-associated mutation in SRSF2 misregulates splicing by altering RNA-binding affinities. Proc. Natl. Acad. Sci. USA.

[54] Komeno Y., Huang Y.-J., Qiu J., Lin L., Xu Y., Zhou Y., Chen L., Monterroza D.D., Li H., DeKelver R.C. (2015). SRSF2 Is Essential for Hematopoiesis, and Its Myelodysplastic Syndrome-Related Mutations Dysregulate Alternative Pre-mRNA Splicing. Mol. Cell. Biol..

[55] Rahman M.A., Lin K.T., Bradley R.K., Abdel-Wahab O., Krainer A.R. (2020). Recurrent SRSF2 Mutations in MDS Affect Both Splicing and NMD. Genes Dev..

[56] DeBoever C., Ghia E.M., Shepard P.J., Rassenti L., Barrett C.L., Jepsen K., Jamieson C.H.M., Carson D., Kipps T.J., Frazer K.A. (2015). Transcriptome Sequencing Reveals Potential Mechanism of Cryptic 3’ Splice Site Selection in SF3B1-mutated Cancers. PLoS Comput. Biol..

[57] Obeng E.A., Chappell R.J., Seiler M., Chen M.C., Campagna D.R., Schmidt P.J., Schneider R.K., Lord A.M., Wang L., Gambe R.G. (2016). Physiologic Expression of Sf3b1K700E Causes Impaired Erythropoiesis, Aberrant Splicing, and Sensitivity to Therapeutic Spliceosome Modulation. Cancer Cell.

[58] Dolatshad H., Pellagatti A., Liberante F.G., Llorian M., Repapi E., Steeples V., Roy S., Scifo L., Armstrong R.N., Shaw J. (2016). Cryptic splicing events in the iron transporter ABCB7 and other key target genes in SF3B1-mutant myelodysplastic syndromes. Leukemia.

[59] Conte S., Katayama S., Vesterlund L., Karimi M., Dimitriou M., Jansson M., Mortera-Blanco T., Unneberg P., Papaemmanuil E., Sander B. (2015). Aberrant splicing of genes involved in haemoglobin synthesis and impaired terminal erythroid maturation in SF3B1 mutated refractory anaemia with ring sideroblasts. Br. J. Haematol..

[60] Visconte V., Avishai N., Mahfouz R., Tabarroki A., Cowen J., Sharghi-Moshtaghin R., Hitomi M., Rogers H.J., Hasrouni E., Phillips J. (2015). Distinct iron architecture in SF3B1-mutant myelodysplastic syndrome patients is linked to an SLC25A37 splice variant with a retained intron. Leukemia.

[61] Furney S.J., Pedersen M., Gentien D., Dumont A.G., Rapinat A., Desjardins L., Turajlic S., Piperno-Neumann S., de la Grange P., Roman-Roman S. (2013). SF3B1 mutations are associated with alternative splicing in uveal melanoma. Cancer Discov..

[62] Inoue D., Chew G.L., Liu B., Michel B.C., Pangallo J., D’Avino A.R., Hitchman T., North K., Lee S.C.W., Bitner L. (2019). Spliceosomal disruption of the non-canonical BAF complex in cancer. Nature.

[63] Graubert T.A., Shen D., Ding L., Okeyo-Owuor T., Lunn C.L., Shao J., Krysiak K., Harris C.C., Koboldt D.C., Larson D.E. (2012). Recurrent mutations in the U2AF1 splicing factor in myelodysplastic syndromes. Nat. Genet..

[64] Przychodzen B., Jerez A., Guinta K., Sekeres M.A., Padgett R., Maciejewski J.P., Makishima H. (2013). Patterns of missplicing due to somatic U2AF1 mutations in myeloid neoplasms. Blood.

[65] Brooks A.N., Choi P.S., De Waal L., Sharifnia T., Imielinski M., Saksena G., Sekhar P.C., Sivachenko A., Rosenberg M., Chmielecki J. (2014). A pan-cancer analysis of transcriptome changes associated with somatic mutations in U2AF1 reveals commonly altered splicing events. PLoS ONE.

[66] Ilagan J.O., Ramakrishnan A., Hayes B., Murphy M.E., Zebari A.S., Bradley P., Bradley R.K. (2015). U2AF1 mutations alter splice site recognition in hematological malignancies. Genome Res..

[67] Okeyo-Owuor T., White B.S., Chatrikhi R., Mohan D.R., Kim S., Griffith M., Ding L., Ketkar-Kulkarni S., Hundal J., Laird K.M. (2015). U2AF1 mutations alter sequence specificity of pre-mRNA binding & splicing. Leukemia.

[68] Yip B.H., Steeples V., Repapi E., Armstrong R.N., Llorian M., Roy S., Shaw J., Dolatshad H., Taylor S., Verma A. (2017). The U2AF1S34Fmutation induces lineage-specific splicing alterations in myelodysplastic syndromes. J. Clin. Investig..

[69] Bejar R., Stevenson K., Abdel-Wahab O., Galili N., Nilsson B.B., Garcia-Manero G., Kantarjian H., Raza A., Levine R.L., Neberg D. (2011). Clinical effect of point mutations in myelodysplastic syndromes. N. Engl. J. Med..

[70] Borel C., Dastugue N., Cances Lauwers V., Mozziconacci M.J., Prebet T., Vey N., Pigneux A., Lippert E., Visanica S., Legrand F. (2012). PICALM-MLLT10 acute myeloid leukemia: A French cohort of 18 patients. Leuk. Res..

[71] Chia N.Y., Chan Y.S., Feng B., Lu X., Orlov Y.L., Moreau D., Kumar P., Yang L., Jiang J., Lau M.S. (2010). A genome-wide RNAi screen reveals determinants of human embryonic stem cell identity. Nature.

[72] Gaspar-Maia A., Qadeer Z.A., Hasson D., Ratnakumar K., Leu N.A., Leroy G., Liu S., Costanzi C., Valle-Garcia D., Schaniel C. (2013). Macro H2A histone variants act as a barrier upon reprogramming towards pluripotency. Nat. Commun..

[73] Nguyen H.D., Yadav T., Giri S., Saez B., Graubert T.A., Zou L. (2017). Functions of replication protein A as a sensor of R loops and a regulator of RNaseH1. Mol. Cell.

[74] Chen L., Chen J.Y., Huang Y.J., Gu Y., Qiu J., Qian H., Shao C., Zhang X., Hu J., Li H. (2018). The augmented R-loop is a unifying mechanism for myelodysplastic syndromes induced by high-risk splicing factor mutations. Mol. Cell.

[75] Park S.M., Ou J., Chamberlain L., Simone T.M., Yang H., Virbasius C.M., Ali A.M., Zhu L.J., Mukherjee S., Raza A. (2016). U2AF35 (S34F) promotes transformation by directing aberrant ATG7 pre-mRNA 3’ end formation. Mol. Cell.

[76] Makishima H., Visconte V., Sakaguchi H., Jankowska A.M., Abu Kar S., Jerez A., Przychodzen B., Bupathi M., Guinta K., Afable M.G. (2012). Mutations in the spliceosome machinery, a novel and ubiquitous pathway in leukemogenesis. Blood.

[77] Quesada V., Conde L., Villamor N., Ordonez G.R., Jares P., Bassaganyas L., Ramsay A.J., Bea S., Pinyol M., Martinez-Trillos A. (2012). Exome sequencing identifies recurrent mutations of the splicing factor SF3B1 gene in chronic lymphocytic leukemia. Nat. Genet..

[78] Walter M.J., Shen D., Shao J., Ding L., White B.S., Kandoth C., Miller C.A., Niu B., McLellan M.D., Dees N.D. (2013). Clonal diversity of recurrently mutated genes in myelodysplastic syndromes. Leukemia.

[79] Seiler M., Peng S., Agrawal A.A., Palacino J., Teng T., Zhu P., Smith P.G., Buonamici S., Yu L., The Cancer Genome Atlas Research Network (2018). Somatic mutational landscape of splicing factor genes and their func-tional consequences across 33 cancer types. Cell Rep..

[80] Madan V., Kanojia D., Li J., Okamoto R., Sato-Otsubo A., Kohlmann A., Sanada M., Grossmann V., Sundaresan J., Shiraishi Y. (2015). Aberrant splicing of U12-type introns is the hallmark of ZRSR2 mutant myelodysplastic syndrome. Nat. Commun..

[81] Ibrahimpasic T., Xu B., Landa I., Dogan S., Middha S., Seshan V., Deraje S., Carlson D.L., Migliacci J., Knauf J.A. (2017). Genomic alterations in fatal forms of nonanaplastic thyroid cancer: Identification of MED12 and RBM10 as novel thyroid cancer genes associated with tumor virulence. Clin. Cancer Res..

[82] Bechara E.G., Sebestyen E., Bernardis I., Eyras E., Valcarcel J. (2013). RBM5, 6, and 10 differentially regulate NUMB alternative splicing to control cancer cell proliferation. Mol. Cell.

[83] Suzuki H., Kumar S.A., Shuai S., Diaz-Navarro A., Gutierrez-Fernandez A., Antonellis P., Cavalli F.M.G., Juraschka K., Farooq H., Shibahara I. (2019). Recurrent noncoding U1 snRNA mutations drive cryptic splicing in SHH medulloblastoma. Nature.

[84] Shuai S., Suzuki H., Diaz-Navarro A., Nadeu F., Kumar S.A., Gutierrez-Fernandez A., Delgado J., Pinyol M., López-Otín C., Puente X.S. (2019). The U1 spliceosomal RNA is recurrently mutated in multiple cancers. Nature.

[85] Anczuków O., Rosenberg A.Z., Akerman M., Das S., Zhan L., Karni R., Muthuswamy S.K., Krainer A.R. (2012). The splicing factor SRSF1 regulates apoptosis and proliferation to promote mammary epithelial cell transformation. Nat. Struct. Mol. Biol..

[86] Zong F.Y., Fu X., Wei W.J., Luo Y.G., Heiner M., Cao L.J., Fang Z., Fang R., Lu D., Ji H. (2014). The RNA-binding protein QKI suppresses cancer-associated aberrant splicing. PLoS Genet..

[87] Goncalves V., Pereira J.F.S., Jordan P. (2017). Signaling pathways driving aberrant splicing in cancer cells. Genes.

[88] Clower C.V., Chatterjee D., Wang Z., Cantley L.C., Vander Heiden M.G., Krainer A.R. (2010). The alternative splicing repressors hnRNP A1/A2 and PTBinfluence pyruvate kinase isoform expression and cell metabolism. Proc. Natl. Acad. Sci. USA.

[89] Das S., Anczukow O., Akerman M., Krainer A.R. (2012). Oncogenic splicing factor SRSF1 is a critical transcriptional target of MYC. Cell Rep..

[90] Ghigna C., Giordano S., Shen H., Benvenuto F., Castiglioni F., Comoglio P.M., Green M.R., Riva S., Biamonti G. (2005). Cell motility is controlled by SF2/ASF through alternative splicing of the Ron protooncogene. Mol. Cell.

[91] Anczuków O., Akerman M., Clery A., Wu J., Shen C., Shirole N.H., Raimer A., Sun S., Jensen M.A., Hua Y. (2015). SRSF1-regulated alternative splicing in breast cancer. Mol. Cell.

[92] Tang Y., Horikawa I., Ajiro M., Robles A.I., Fujita K., Mondal A.M., Stauffer J.K., Zheng Z.M., Harris C.C. (2013). Downregulation of splicing factor SRSF3 induces p53β, an alternatively spliced isoform of p53 that promotes cellular senescence. Oncogene.

[93] Rauch J., Moran-Jones K., Albrecht V., Schwarzl T., Hunter K., Gires O., Kolch W. (2011). c-Myc regulates RNA splicing of the A-Raf kinase and its activation of the ERK pathway. Cancer Res..

[94] Xiao S.H., Manley J.L. (1997). Phosphorylation of the ASF/SF2 RS domain affects both protein-protein and protein-RNA interactions and is necessary for splicing. Genes Dev..

[95] Tazi J., Kornstadt U., Rossi F., Jeanteur P., Cathala G., Brunel C., Lührmann R. (1993). Thiophosphorylation of U1-70K protein inhibits pre-mRNA splicing. Nature.

[96] Cao W., Jamison S.F., Garcia-Blanco M.A. (1997). Both phosphorylation and dephosphorylation of ASF/SF2 are required for pre-mRNA splicing in vitro. RNA.

[97] Rossi F., Labourier E., Forne T., Divita G., Derancourt J., Riou J.F., Antoine E., Cathala G., Brunel C., Tazi J. (1996). Specific phosphorylation of SR proteins by mammalian DNA topoisomerase I. Nature.

[98] Blaustein M., Pelisch F., Coso O.A., Bissell M.J., Kornblihtt A.R., Srebrow A. (2004). Mammary epithelial-mesenchymal interaction regulates fibronectin alternative splicing via phosphatidylinositol 3-kinase. J. Biol. Chem..

[99] Zhou Z., Qiu J., Liu W., Zhou Y., Plocinik R.M., Li H., Hu Q., Ghosh G., Adams J.A., Rosenfeld M.G. (2012). The Akt-SRPK-SR axis constitutes a major pathway in transducing EGF signaling to regulate alternative splicing in the nucleus. Mol. Cell.

[100] Hayes G.M., Carrigan P.E., Beck A.M., Miller L.J. (2006). Targeting the RNA splicing machinery as a novel treatment strategy for pancreatic carcinoma. Cancer Res..

[101] Hayes G.M., Carrigan P.E., Miller L.J. (2007). Serine-arginine protein kinase 1 overexpression is associated with tumorigenic imbalance in mitogen-activated protein kinase pathways in breast, colonic, and pancreatic carcinomas. Cancer Res..

[102] Palacino J., Swalley S.E., Song C., Cheung A.K., Shu L., Zhang X., Van Hoosear M., Shin Y., Chin D.N., Keller C.G. (2015). SMN2 splice modulators enhance U1-pre-mRNA association and rescue SMA mice. Nat. Chem. Biol..

[103] Krishnakumar S., Mohan A., Kandalam M., Ramkumar H.L., Venkatesan N., Das R.R. (2008). SRPK1: A cisplatin sensitive protein expressed in retinoblastoma. Pediatr. Blood Cancer.

[104] Amin E.M., Oltean S., Hua J., Gammons M.V., Hamdollah-Zadeh M., Welsh G.I., Cheung M.K., Ni L., Kase S., Rennel E.S. (2011). WT1 mutants reveal SRPK1 to be a downstream angiogenesis target by altering VEGF splicing. Cancer Cell.

[105] Goehe R.W., Shultz J.C., Murudkar C., Usanovic S., Lamour N.F., Massey D.H., Zhang L., Camidge D.R., Shay J.W., Minna J.D. (2010). hnRNP L regulates the tumorigenic capacity of lung cancer xenografts in mice via caspase-9 pre-mRNA processing. J. Clin. Investig..

[106] Vu N.T., Park M.A., Shultz J.C., Goehe R.W., Hoeferlin L.A., Shultz M.D., Smith S.A., Lynch K.W., Chalfant C.E. (2013). hnRNP U enhances caspase-9 splicing and is modulated by AKT-dependent phosphorylation of hnRNP L. J. Biol. Chem..

[107] Jo O.D., Martin J., Bernath A., Masri J., Lichtenstein A., Gera J. (2008). Heterogeneous nuclear ribonucleoprotein A1 regulates cyclin D1 and c-myc internal ribosome entry site function through Akt signaling. J. Biol. Chem..

[108] Bonnal S., Vigevani L., Valcárcel J. (2012). The spliceosome as a target of novel antitumour drugs. Nat. Rev. Drug Discov..

[109] Webb T.R., Joyner A.S., Potter P.M. (2013). The development and application of small molecule modulators of SF3b as therapeutic agents for cancer. Drug Discov. Today.

[110] Yokoi A., Kotake Y., Takahashi K., Kadowaki T., Matsumoto Y., Minoshima Y., Sugi N.H., Sagane K., Hamaguchi M., Iwata M. (2011). Biological validation that SF3b is a target of the antitumor macrolide pladienolide. FEBS J..

[111] Teng T., Tsai J.H., Puyang X., Seiler M., Peng S., Prajapati S., Aird D., Buonamici S., Caleb B., Chan B. (2017). Splicing modulators act at the branch point adenosine binding pocket defined by the PHF5A-SF3b complex. Nat. Commun..

[112] Finci L.I., Zhang X., Huang X., Zhou Q., Tsai J., Teng T., Agrawal A., Chan B., Irwin S., Karr C. (2018). The cryo-EM structure of the SF3b spliceosome complex bound to a splicing modulator reveals a pre-mRNA substrate competitive mechanism of action. Genes Dev..

[113] Cretu C., Agrawal A.A., Cook A., Will C.L., Fekkes P., Smith P.G., Lührmann R., Larsen N., Buonamici S., Pena V. (2018). Structural basis of splicing modulation by antitumor macrolide compounds. Mol. Cell.

[114] Eskens F.A., Ramos F.J., Burger H., O’Brien J.P., Piera A., de Jonge M.J., Mizui Y., Wiemer E.A., Carreras M.J., Baselga J. (2013). Phase I, pharmacokinetic and pharmacodynamic study of the first- in-class spliceosome inhibitor E7107 in patients with advanced solid tumors. Clin. Cancer Res..

[115] Hong D.S., Kurzrock R., Naing A., Wheler J.J., Falchook G.S., Schiffman J.S., Faulkner N., Pilat M.J., O’Brien J., LoRusso P. (2014). A phase I, open- label, single- arm, dose- escalation study of E7107, a precursor messenger ribonucleic acid (pre-mRNA) splicesome inhibitor administered intravenously on days 1 and 8 every 21 days to patients with solid tumors. Investig. New Drugs.

[116] Mizui Y., Sakai T., Iwata M., Uenaka T., Okamoto K., Shimizu H., Yamori T., Yoshimatsu K., Asada M. (2004). Pladienolides, new substances from culture of Streptomyces platensis Mer-11107. III. In vitro and in vivo antitumor activities. J. Antibiot..

[117] Sakai T., Asai N., Okuda A., Kawamura N., Mizui Y. (2004). Pladienolides, new substances from culture of Streptomyces platensis Mer-11107. II. Physico-chemical properties and structure elucidation. J. Antibiot..

[118] Salton M., Misteli T. (2016). Small molecule modulators of pre-mRNA splicing in cancer therapy. Trends Mol. Med..

[119] Kotake Y., Sagane K., Owa T., Mimori-Kiyosue Y., Shimizu H., Uesugi M., Ishihama Y., Iwata M., Mizui Y. (2007). Splicing factor SF3b as a target of the antitumor natural product pladienolide. Nat. Chem. Biol..

[120] Iwata M., Ozawa Y., Uenaka T., Shimizu H., Niijima J., Kanada R.M., Fukuda Y., Nagai M., Kotake Y., Yoshida M. (2004). E7107, a new 7-urethane derivative of pladienolide D, displays curative effect against several human tumor xenografts. Cancer Res..

[121] Nakajima H., Hori Y., Terano H., Okuhara M., Manda T., Matsumoto S., Shimomura K. (1996). New antitumor substances, FR901463, FR901464 and FR901465. II. Activities against experimental tumors in mice and mechanism of action. J. Antibiot..

[122] Nakajima H., Sato B., Fujita T., Takase S., Terano H., Okuhara M. (1996). New antitumor substances, FR901463, FR901464 and FR901465. I. Taxonomy, fermentation, isolation, physicochemical properties and biological activities. J. Antibiot..

[123] Uehara T., Minoshima Y., Sagane K., Sugi N.H., Mitsuhashi K.O., Yamamoto N., Kamiyama H., Takahashi K., Kotake Y., Uesugi M. (2017). Selective degradation of splicing factor CAPERalpha by anticancer sulfonamides. Nat. Chem. Biol..

[124] Han T., Goralski M., Gaskill N., Capota E., Kim J., Ting T.C., Xie Y., Williams N.S., Niijhawan D. (2017). Anticancer sulfonamides target splicing by inducing RBM39 degradation via recruitment to DCAF15. Science.

[125] Obeng E.A., Stewart C., Abdel-Wahab O. (2019). Altered RNA processing in cancer pathogenesis and therapy. Cancer Discov..

[126] Hatcher J.M., Wu G., Zeng C., Zhu J., Meng F., Patel S., Wang W., Ficarro S.B., Leggett A.L., Powell C.E. (2018). SRPKIN-1: A covalent SRPK1/2 inhibitor that potently converts VEGF from proangiogenic to anti- angiogenic isoform. Cell Chem. Biol..

[127] Fukuhara T., Hosoya T., Shimizu S., Sumi K., Oshiro T., Yoshinaka Y., Suzuki M., Yamamoto N., Herzenberg L.A., Herzenberg L.A. (2006). Utilization of host SR protein kinases and RNA splicing machinery during viral replication. Proc. Natl. Acad. Sci. USA.

[128] Desterro J., Bak-Gordon P., Carmo-Fonseca M. (2020). Targeting mRNA processing as an anticancer strategy. Nat. Rev. Drug Discov..

[129] Muraki M., Ohkawara B., Hosoya T., Onogi H., Koizumi J., Koizumi T., Sumi K., Yomoda J., Murray M.V., Kimura H. (2004). Manipulation of alternative splicing by a newly developed inhibitor of Clks. J. Biol. Chem..

[130] Iwai K., Yaguchi M., Nishimura K., Yamamoto Y., Tamura T., Nakata D., Dairiki R., Kawakita Y., Mizojiri R., Ito Y. (2018). Anti-tumor efficacy of a novel CLK inhibitor via targeting RNA splicing and MYC-dependent vulnerability. EMBO Mol. Med..

[131] Araki S., Dairiki R., Nakayama Y., Murai A., Miyashita R., Iwatani M., Nomura T., Nakanishi O. (2015). Inhibitors of CLK protein kinases suppress cell growth and induce apoptosis by modulating pre-mRNA splicing. PLoS ONE.

[132] Pilch B., Allemand E., Facompré M., Bailly C., Riou J.F., Soret J., Tazi J. (2001). Splicing inhibition of serine- and arginine-rich splicing factors phosphorylation, spliceosome assembly, and splicing by the antitumor drug NM-506. Cancer Res..

[133] Chang J.G., Yang D.M., Chang W.H., Chow L.P., Chan W.L., Lin H.H., Huang H.D., Chang Y.S., Hung C.H., Yang W.K. (2011). Small molecule amiloride modulates oncogenic RNA alternative splicing to devitalize human cancer cells. PLoS ONE.

[134] Denichenko P., Mogilevsky M., Cléry A., Welte T., Biran J., Shimshon O., Barnabas G.D., Danan-Gotthold M., Kumar S., Yavin E. (2019). Specific inhibition of splicing factor activity by decoy RNA oligonucleotides. Nat. Commun..

[135] Lee S.C., North K., Kim E., Jang E., Obeng E., Lu S.X., Liu B., Inoue D., Yoshimi A., Ki M. (2018). Synthetic lethal and convergent biological effects of cancer-associated spliceosomal gene mutations. Cancer Cell.

[136] Lee S.C., Dvinge H., Kim E., Cho H., Micol J.B., Chung Y.R., Durham B.H., Yoshimi A., Kim Y.J., Thomas M. (2016). Modulation of splicing catalysis for therapeutic targeting of leukemia with mutations in genes encoding spliceosomal proteins. Nat. Med..

[137] Shirai C.L., Tripathi M., Ley J.N., Ndonwi M., White B.S., Tapia R., Saez B., Bertino A., Shao J., Kim S. (2015). Preclinical activity of splicing modulators in U2AF1-mutant MDS–AML. Blood.

[138] Assi R., Kantarjian H.M., Kadia T.M., Pemmaraju N., Jabbour E., Jain N., Daver N., Estrov Z., Uehara T., Owa T. (2018). Final results of a phase 2, open-label study of indisulam, idarubicin, and cytarabine in patients with relapsed or refractory acute myeloid leukemia and high-risk myelodysplastic syndrome. Cancer.

[139] Talbot D.C., von Pawel J., Cattell E., Yule S.M., Johnston C., Zandvliet A.S., Huitema A.D., Norbury C.J., Ellis P., Bosquee L. (2007). A randomized phase II pharmacokinetic and pharmacodynamics study of indisulam as second-line therapy in patients with advanced non-small cell lung cancer. Clin. Cancer Res..

[140] Supuran C.T. (2003). Indisulam: An anticancer sulfonamide in clinical development. Expert Opin. Investig. Drugs.

[141] Wang E., Lu S.X., Pastore A., Chen X., Imig J., Chun-Wei Lee S., Hockemeyer K., Ghebrechristos Y.R., Yoshimi A., Inoue D. (2019). Targeting an RNA-binding protein network in acute myeloid leukemia. Cancer Cell.

[142] Jiang L., Huang J., Higgs B.W., Hu Z., Xiao Z., Yao X., Conley S., Zhong H., Liu Z., Brohawn P. (2016). Genomic landscape survey identifies SRSF1 as a key oncodriver in small cell lung cancer. PLoS Genet..

[143] Shepard P.J., Hertel K.J. (2009). The SR protein family. Genome Biol..

[144] Busch A., Hertel K.J. (2012). Evolution of SR protein and hnRNP splicing regulatory factors. Wiley Interdiscip. Rev. RNA.

[145] Giannakouros T., Nikolakaki E., Mylonis I., Georgatsou E. (2011). Serine–arginine protein kinases: A small protein kinase family with a large cellular presence. FEBS J..

[146] Zhou Z., Fu X.D. (2013). Regulation of splicing by SR proteins and SR protein-specific kinases. Chromosoma.

[147] McClorey G., Wood M.J. (2015). An overview of the clinical application of antisense oligonucleotides for RNA-targeting therapies. Curr. Opin. Pharmacol..

[148] Meister G., Eggert C., Buhler D., Brahms H., Kambach C., Fischer U. (2001). Methylation of Sm proteins by a complex containing PRMT5 and the putative U snRNP assembly factor pICln. Curr. Biol..

[149] Brahms H., Meheus L., de Brabandere V., Fischer U., Lührmann R. (2001). Symmetrical dimethylation of arginine residues in spliceosomal Sm protein B/B’ and the Sm-like protein LSm4, and their interactionwith the SMN protein. RNA.

[150] Hsu T.Y., Simon L.M., Neill N.J., Marcotte R., Sayad A., Bland C.S., Echeverria G.V., Sun T., Kurley S.J., Tyagi S. (2015). The spliceosome is a therapeutic vulnerability in MYC-driven cancer. Nature.

[151] Banasavadi-Siddegowda Y.K., Welker A.M., An M., Yang X., Zhou W., Shi G., Imitola J., Li C., Hsu S., Wang J. (2018). PRMT5 as a druggable target for glioblastoma therapy. Neuro. Oncol..

[152] Yang Y., Bedford M.T. (2013). Protein arginine methyltransferases and cancer. Nat. Rev. Cancer.

[153] Zhang L., Tran N.T., Su H., Wang R., Lu Y., Tang H., Aoyagi S., Guo A., Khodadadi-Jamayran A., Zhou D. (2015). Cross-talk between PRMT1-mediated methylation and ubiquitylation on RBM15 controls RNA splicing. eLife.

[154] Gao G., Zhang L., Villarreal O.D., He W., Su D., Bedford E., Moh P., Shen J., Shi X., Bedford M.T. (2019). PRMT1 loss sensitizes cells to PRMT5 inhibition. Nucleic Acids Res..

[155] Koh C.M., Bezzi M., Low D.H., Ang W.X., Teo S.X., Gay F.P., Al-Haddawi M., Tan S.Y., Osato M., Sabò A. (2015). MYC regulates the core pre-mRNA splicing machinery as an essential step in lymphomagenesis. Nature.

[156] Kryukov G.V., Wilson F.H., Ruth J.R., Paulk J., Tsherniak A., Marlow S.E., Vazquez F., Weir B.A., Fitzgerald M.E., Tanaka M. (2016). MTAP deletion confers enhanced dependency on the PRMT5 arginine methyltransferase in cancer cells. Science.

[157] Mavrakis K.J., McDonald E.R., Schlabach M.R., Billy E., Hoffman G.R., de Weck A., Ruddy D.A., Venkatesan K., Yu J., McAllister G. (2016). Disordered methionine metabolism in MTAP- and CDKN2A-deleted cancers leads to dependence on PRMT5. Science.

[158] Vu L.P., Cheng Y., Kharas M.G. (2019). The biology of m(6)A RNA methylation in normal and malignant hematopoiesis. Cancer Discov..

[159] Yamauchi T., Masuda T., Canver M.C., Seiler M., Semba Y., Shboul M., Al-Raqad M., Maeda M., Schoonenberg V.A.C., Cole M.A. (2018). Genome-wide CRISPR-Cas9 screen identifies leukemia-specific dependence on a pre-mRNA metabolic pathway regulated by DCPS. Cancer Cell.

[160] Gogliotti R.G., Cardona H., Singh J., Bail S., Emery C., Kuntz N., Jorgensen M., Durens M., Xia B., Barlow C. (2013). The DcpS inhibitor RG3039 improves survival, function and motor unit pathologies in two SMA mouse models. Hum. Mol. Genet..

[161] Kole R., Krainer A.R., Altman S. (2012). RNA therapeutics: Beyond RNA interference and antisense oligonucleotides. Nat. Rev. Drug Discov..

[162] Rigo F., Hua Y., Krainer A.R., Bennett C.F. (2012). Antisense-based therapy for the treatment of spinal muscular atrophy. J. Cell Biol..

[163] Bennett C.F. (2019). Therapeutic antisense oligonucleotides are coming of age. Annu. Rev. Med..

[164] Charleston J.S., Schnell F.J., Dworzak J., Donoghue C., Lewis S., Chen L., Young G.D., Milici A.J., Voss J., DeAlwis U. (2018). Eteplirsen treatment for Duchenne muscular dystrophy: Exon skipping and Dystrophin production. Neurology.

[165] Hua Y., Vickers T.A., Okunola H.L., Bennett C.F., Krainer A.R. (2008). Antisense masking of an hnRNP A1/A2 intronic splicing silencer corrects SMN2 splicing in transgenic mice. Am. J. Hum. Genet..

[166] Michelson D., Ciafaloni E., Ashwal S., Lewis E., Narayanaswami P., Oskoui M., Armstrong M.J. (2018). Evidence in focus: Nusinersen use in spinal muscular atrophy: Report of the Guideline Development, Dissemination, and Implementation Subcommittee of the American Academy of Neurology. Neurology.

[167] Wang Z., Jeon H.Y., Rigo F., Bennett C.F., Krainer A.R. (2012). Manipulation of PK-M mutually exclusive alternative splicing by antisense oligonucleotides. Open Biol..

[168] Hastings M.L., Berniac J., Liu Y.H., Abato P., Jodelka F.M., Barthel L., Kumar S., Dudley C., Nelson M., Larson K. (2009). Tetracyclines that promote SMN2 exon 7 splicing as therapeutics for spinal muscular atrophy. Sci. Transl. Med..

[169] Naryshkin N.A., Weetall M., Dakka A., Narasimhan J., Zhao X., Feng Z., Ling K.K., Karp G.M., Qi H., Woll M.G. (2014). Motor neuron disease. SMN2 splicing modifiers improve motor function and longevity in mice with spinal muscular atrophy. Science.

[170] Yoshida M., Kataoka N., Miyauchi K., Ohe K., Iida K., Yoshida S., Nojima T., Okuno Y., Onogi H., Usui T. (2015). Rectifier of aberrant mRNA splicing recovers tRNA modification in familial dysautonomia. Proc. Natl. Acad. Sci. USA.

[171] Ott P.A., Hu Z., Keskin D.B., Shukla S.A., Sun J., Bozym D.J., Zhang W., Luoma A., Giobbie-Hurder A., Peter L. (2017). An immunogenic personal neoantigen vaccine for patients with melanoma. Nature.

[172] Sahin U., Derhovanessian E., Miller M., Kloke B.P., Simon P., Löwer M., Bukur V., Tadmor A.D., Luxemburger U., Schrörs B. (2017). Personalized RNA mutanome vaccines mobilize poly-specific therapeutic immunity against cancer. Nature.

[173] Bhattacharjee S., Nandi S. (2016). Choices have consequences: The nexus between DNA repair pathways and genomic instability in cancer. Clin. Transl. Med..

[174] Bhattacharjee S., Nandi S. (2017). Synthetic lethality in DNA repair network: A novel avenue in targeted cancer therapy and combination therapeutics. IUBMB Life.

[175] Bhattacharjee S., Nandi S. (2017). DNA damage response and cancer therapeutics through the lens of the Fanconi Anemia DNA repair pathway. Cell Commun. Signal..

[176] Bhattacharjee S., Nandi S. (2018). Rare Genetic Diseases with Defects in DNA Repair: Opportunities and Challenges in Orphan Drug Development for Targeted Cancer Therapy. Cancers.

[177] Ghosh D., Venkatramani P., Nandi S., Bhattacharjee S. (2019). CRISPR–Cas9 a boon or bane: The bumpy road ahead to cancer therapeutics. Cancer Cell Int..

[178] Huilgol D., Venkataramani P., Nandi S., Bhattacharjee S. (2019). Targeting transcription factors that govern development and disease: An achilles heel for cancer therapeutics. Genes.

[179] Kim Y.J., Rahman M.A. (2018). NMD in diseases and potential therapies. J. Investig. Genom..

[180] Nazim M., Nasrin F., Rahman M.A. (2018). Coordinated regulation of alternative splicing and alternative polyadenylation. J. Genet. Genet. Eng..

